# Decadal shifts in traits of reef fish communities in marine reserves

**DOI:** 10.1038/s41598-021-03038-9

**Published:** 2021-12-06

**Authors:** Jeneen Hadj-Hammou, Tim R. McClanahan, Nicholas A. J. Graham

**Affiliations:** 1 Lancaster University Environment Centre, Lancaster University, Lancaster, UK; 2 Wildlife Conservation Society, Global Marine Programs, Bronx, NY 10460 USA

**Keywords:** Community ecology, Conservation biology

## Abstract

Marine reserves are known to impact the biomass, biodiversity, and functions of coral reef fish communities, but the effect of protective management on fish traits is less explored. We used a time-series modelling approach to simultaneously evaluate the abundance, biomass, and traits of eight fish families over a chronosequence spanning 44 years of protection. We constructed a multivariate functional space based on six traits known to respond to management or disturbance and affect ecosystem processes: size, diet, position in the water column, gregariousness, reef association, and length at maturity. We show that biomass increased with a log-linear trend over the time-series, but abundance only increased after 20 years of closure, and with more variation among reserves. This difference is attributed to recovery rates being dependent on body sizes. Abundance-weighted traits and the associated multivariate space of the community change is driven by increased proportions over time of the trait categories: 7–15 cm body size; planktivorous; species low in the water column; medium-large schools; and species with high levels of reef association. These findings suggest that the trait compositions emerging after the cessation of fishing are novel and dynamic.

## Introduction

No-take marine reserves are a widely used management and conservation tool, the implementation of which has been linked to a range of outcomes including increases in fish abundance, biomass, diversity, and the presence of functionally important species^1–3^. Quantifying the trajectories of key groups of organisms in reserves can help identify the mechanisms driving community-level responses^4,5^. However, variability in the temporal trends of traits and how they relate to community biomass and abundance in marine reserves remains largely unexplored. Looking at such temporal trends can often point to useful information about the response of ecosystem functional potential to conservation measures^6^, with traits sometimes responding earlier than taxonomic measures^7^.

Functional approaches to conservation prioritise the maintenance of ecosystem functions and services of highly diverse ecosystems in the dynamic and changing world of the Anthropocene^8^. Ecosystem functioning can be measured directly as the rates of an ecosystem process (e.g., herbivory, predation, bioerosion, nutrient cycling) or indirectly as the functional potential of the ecosystem by looking at the functional groups or traits present within a community^9^. While only indirectly capturing ecological processes, traits are more available in literature compilations and therefore can be applied to datasets retrospectively^10^.

“Functional traits” are suggestive of the mechanistic links between species’ responses to disturbances and management practices and their potential effects on ecosystem processes^11^. The first step in applying a trait-based approach is therefore to carefully select the traits most applicable to the ecological processes and research questions of interest. Trait selection is important for understanding the pathways of community responses and their associated implications^12^. When assessing the functional structure of a community, traits can be weighted by abundance or biomass, allowing for proportional representation^13,14^, with abundance-weighting common practice in broad trait-based approaches^15^.

Changes in species and traits with time since protection can produce novel functional configurations. Such novel configurations can sometimes produce the same ecosystem processes as previous communities, result in the loss of some functioning, or a new balance of functions and services can establish^16–18^. Key traits such as fish body size, trophic level, and life history strategies mediate the relationship between disturbance/recovery and abundance, biomass, and biomass production—all essential components for sustainable ecosystem functioning^19–23^.

An assessment of changes over time in the traits of coral reef fish following establishment of marine reserves would enable a better understanding of the indicative impacts of protection on ecosystem functioning. In this paper, we apply a trait-based approach to a unique long-term dataset on high-compliance no-take marine reserves in Kenya, enabling a range of theory-based predictions to be evaluated (Supplementary Table S1). Specifically, we ask:

1. Do biomass and abundance trends vary over time in marine reserves?
2. Does the abundance-based trait-space of the fish community change over time in marine reserves?
3. Do the relative abundances of individual trait categories progressively shift over time in marine reserves?

## Materials and methods

### Study sites

Kenya has four high compliance no-take marine reserves. Each of the reserves are regularly patrolled government national parks and differ in when they were legally established. Malindi Marine Park is the oldest reserve and was created in 1968, followed by Watamu Marine Park in 1972, Kisite Marine Park in 1973, and Mombasa Marine Park in 1991 (see map in^24^). The sizes of the reserves’ closures vary. Mombasa is 6 km^2^, Malindi is 6.3 km^2^, Watamu is 10 km^2^, and Kisite is 28 km^2^; however, the amount of coral reef area within Kisite Marine park is ~ 10 km^2^. Thus, the range in effective coral reef protected area is 6–10 km^2^^25^. Malindi and Watamu are situated in close proximity. Malindi was excluded from the analyses of this study, because it was severely impacted by the 1998 bleaching event, with the fish community following lagged trends in benthic condition (Supplementary Fig. S2). Inclusion would bias the results towards benthic influence^26^. For the purposes of this paper, we were more interested in the effect of protection from fishing on the fish community, and as explained below, treated the reserves as a chronosequence (see “Marine protection chronosequence” section). The remaining three marine reserves provide a powerful dataset, spanning 44 years of protection from fishing and 732 ecological surveys.

### Fish and benthic surveying

Visual censuses of fish were conducted by the same observer (TRM) during neap tides along two to five 5 × 100 m belt transects in each site. All surveyed sites in the parks were located in the shallow back-reef lagoon or leeward areas. Eight fish families were sampled at species level with abundance counted consistently across the full duration of monitoring from 1991 to 2018: Acanthuridae, Balistidae, Chaetodontidae, Diodontidae, Labridae (including Scarinae), Monacanthidae, Pomacanthidae, and Pomacentridae. These families include all of the trait categories explored in this analysis. However, some trait categories were less well represented than others, namely piscivores, pelagic species, and species with low levels of reef association. Species were counted using a discrete group sampling (DGS) method, whereby families or species with similar body shapes or behaviours were identified and counted during separate passes along a transect^24,27^. Total fish abundances (as well as trait-level abundances) were calculated as the mean number of fish/transect and standardised to the mean number of fish/ha. DGS survey dates and sites are presented in Supplementary Table S7. Benthic surveys were conducted on 9–27 10 m line transects at each site using the line-intercept method. Distances of benthic cover categories under the line were assigned to nine groups: hard coral, soft coral, algal turf, coralline algae, calcareous algae, fleshy algae, seagrass, sand, and sponge.

Biomass was estimated using a different method whereby fish were surveyed at the family level within two to six 5 × 100 m belt transects in each site (see^28^ for further explanation of the two methods). Total lengths of individual fish were estimated and grouped into 10-cm size-class intervals. Total wet mass was estimated for each size-class using established length-mass relationships based on the centre point of the size-classes^29^. The families sampled in the species level abundance counts and used in the biomass analyses represented 74.2% of total biomass (in 2018). For the biomass over time model, individual site-year biomass values were used.

### Fish traits

Seven species-level fish traits were evaluated in this paper: body length (size), diet, schooling behaviour (gregariousness), position in the water column, reef association, and length at maturity. These traits were carefully selected according to whether they were likely to respond to protection from fishing and affect ecosystem functioning^11^ (see trait inclusion justification; Supplementary Table S1). The trait-based analysis was based on abundance data, as species level biomass estimates were not possible from the survey methodology, and the literature on trait-based ecology favours abundance-weighting^15^. Trait values were obtained from the Gaspar database^30^, Fishbase^31^, and FishLife^32^. Data were available for 216 out of 219 species surveyed in the nine families; therefore, three species were excluded from the analyses.

### Data analysis

#### Marine protection chronosequence

To assess how the abundance, biomass, and functional space of the fish community changed over time with protection, the temporal parameter “time since closure” was derived for each of the marine reserves. This was done for each sample point within each reserve by calculating the number of years since the establishment of the marine reserve (the year of data collection minus the year at which the marine reserve was established) to assemble a chronosequence of the data. This method has been applied to the same data to create a time-series spanning several decades of marine protection^28^.

#### Functional space

A functional space based on fish traits within the marine reserves was constructed by carrying out a Principal Coordinates Analysis (PCoA). The PCoA was based on a Gower’s distance matrix of species-level fish traits (size, diet, gregariousness, position in the water column, reef association, and length at maturity) for all years and sites using the R packages, “cluster”^33^ and “ape”^34^. An abundance-weighted mean PCoA value for axes one and two was calculated for each site/year combination. A Pearson’s correlation analysis between PCoA axes 1 and 2 values and community weighted mean (CWM) trait values shows the extent to which each of the traits were associated with the axes.

CWM trait values were calculated for each trait using the “FD” package^35^ as:

$$CWM = \mathop \sum \limits_{z = 1}^{n} p_{z} x_{z}$$

where the site-level abundance of a species *z* in a given year is denoted as *p*_*z*_, and *x*_*z*_ is the trait value of species *z*^36^. For each categorical or ordinal trait, the proportion of trait categories within a trait was calculated as:

$$proportional\_abundance\;i = \frac{\sum Abundance \;of\;species\;with\;attribute\;class\;i}{{\sum Abundance\;of\;all\;species}}$$

The proportional abundance of individual traits over time were weighted by total abundance in each sampling unit. For the continuous trait, length at maturity, the abundance-weighted mean value of that trait was modelled.

#### Covariates

Several covariates explaining variation in the trait space (Supplementary Fig. S6) were included in the global models. The first covariate controlled for in the models represented the benthic community of the sites. A Principal Component Analysis (PCA) was conducted on percentage cover of (1) hard coral, (2) macroalgae, (3) coralline algae, and (4) other calcareous algae across all sites. This produced a succinct multivariate value (PCA axis 1 explaining 50% of the variation) for each site/year that captured multiple aspects of the benthos and at the same time reduced the number of parameters needed to be included in the models. Rugosity, a measure of the structural complexity of the reef^37^, was included as a covariate in the models separate to the PCA of the benthic community. The mean biomass (of the eight fish families) for each marine reserve per year was also calculated and used as a covariate. For years and sites where fish survey data were collected, but other covariate data (e.g. benthic, rugosity, biomass) were missing at random points across the time-series, a Generalized Additive Mixed Model (GAMM) of the covariate over time (calendar year), with reserve as a random effect, was conducted to impute missing data from fitted values. The models were fit with a Gaussian error distribution and followed model validation protocol described below.

The next covariate incorporated into the models was a time-series of Thermal Stress Anomalies (TSAs). TSAs were included in the models as they were associated with coral bleaching events. Moreover, McClanahan^38^ showed that variation in TSA is associated with the biomass of certain fish families. Fish communities were expected to exhibit a lagged response to disturbances such as thermal stress^14^. TSA data from 1991 to 2018 for each marine park were extracted from The Coral Reef Temperature Anomaly Database (CoRTAD) hosted by NOAA Coral Reef Watch. TSAs were calculated for 4 km grid cells as the weekly sea surface temperature minus the maximum weekly climatological (long-term average) sea surface temperature^39^. The maximum TSA (magnitude) for each reserve in each year was selected for modelling. Therefore, the optimal time-lag for the effect of TSAs on fish functional space was assessed by lagging TSA values from 0 to 9 years and incorporating this lag into a GAMM model of the first PCoA axis. Lagged models were compared (for the same dataset years), and an optimal-fit lag of 4 years was selected to be included in the models, using the AIC selection procedure described below. The Granger Test, convergent cross-mapping and cross-correlation methods of detecting causality and time-lagged effects of covariates were trialled^14,40^. However, due to uneven time-steps in the time-series, a modelling approach for selecting the optimal thermal stress time-lag was favoured (e.g.^41^). The 4-year lag fits with previous findings showing that coral cover took approximately 4 years to return close to pre-1998 bleaching levels^42^.

Oceanic productivity was estimated using chlorophyll a for the years 1997–2018, which were available from the Ocean Colour Climate Change Initiative dataset esa-cci-chla-monthly-v4-1 by the European Space Agency (http://www.esa-oceancolour-cci.org/). Daily data were averaged to get annual values at a 4-km resolution. For years prior to 1997, the average value of chlorophyll a for each park over the time-series was taken. Net Primary Productivity (NPP), another measure of oceanic productivity, was obtained as a static average value for the centre of each park from the Marine Socio-Environmental Covariates database^43^.

An initial set of covariates including time since closure, calendar year, axis 1 of a benthic PCA, rugosity, chlorophyll a, NPP, TSA, and biomass were tested for collinearity using VIF values and checking the correlation matrices^44^. Biomass, calendar year, and NPP had VIF values > 3 and were therefore removed from models. All continuous covariates were scaled and centred to a mean of zero and standard deviation of one for model fitting.

Two modelling approaches were taken to explore community changes in biomass, abundance, and functional space (PCoA) over time. The first approach was to include the marine reserve (Mombasa, Kisite, Watamu) as a random effect. The second approach was to allow slopes and intercepts to vary by marine reserve. In applying these two approaches, we illustrate how the reserves form continuous patterns across the chronosequence and where they differ. For illustration purposes, all covariates aside from time since closure were held to their means, and partial residuals that account for covariate effects in the models (rather than raw data points) were presented. A summary of covariates can be found in Supplementary Table S2.

#### Modelling

All research questions were addressed using GAMMs with the R package “mgcv”^45^ to model changes of respective variables of interest over time since closure of the marine parks. GAMMs were favoured over other modelling tools, because they allow for the detection of non-linear patterns discovered in this dataset with exploratory analyses and typically present in time-series data^46^. A backwards selection process, whereby each variable was eliminated until all variables left in the model were significant (*p* < 0.05), was used to select the optimal model, as determined by AIC scores (optimal model < 2 AIC from other models). Where models did not differ more than 2 AIC, the simplest model with the fewest parameters was selected. Smoother functions for continuous covariates were fit with cubic regression splines^47^. The number of knots (k) in a smoother determines the “wiggliness” of the smoother parameter’s curve^48^. This number was estimated by comparing Estimated Degrees of Freedom (EDF) values to k and through a generalized cross validation technique. The number of knots was restricted to four for the time since closure parameter, in order to allow for polynomial relationships and to detect a range of non-linear trends, but also to restrain the flexibility of model fits for ease of interpretation and to limit computation time^49^. An ARMA(1,0) residual autocorrelation structure was added to the models to account for dependent values on preceding years within the same site^50^. The need for an autocorrelation structure was assessed visually using the autocorrelation function (ACF)^44^. For the biomass model with the marine reserve as a random effect, a residual variation structure, VarPower, was also incorporated. Optimal model equations and outputs can be found in Supplementary Tables S3–S5.

All models were validated following protocols outlined in Zuur and Ieno^44^. Significant outliers, as determined by Cook’s Distance, were removed to ensure they did not over-influence results^44^ (but see Supplementary Fig. S5). Model assumptions were checked by plotting model residuals against fitted values as well as covariates included and excluded from the models. Biomass and abundance data were log-transformed and trait proportions were logit-transformed in order to normalise the residuals (as in^49,51^), after trialling the use of different distribution families^52^. Biomass, abundance, and proportional trait models had a Gaussian distributed error term, while PCoA and length at maturity (logged cm) had a Gamma distributed error term. All data analysis was conducted using R Version 3.6.3.^53^.

## Results

### Biomass and abundance models

**Figure 1:**
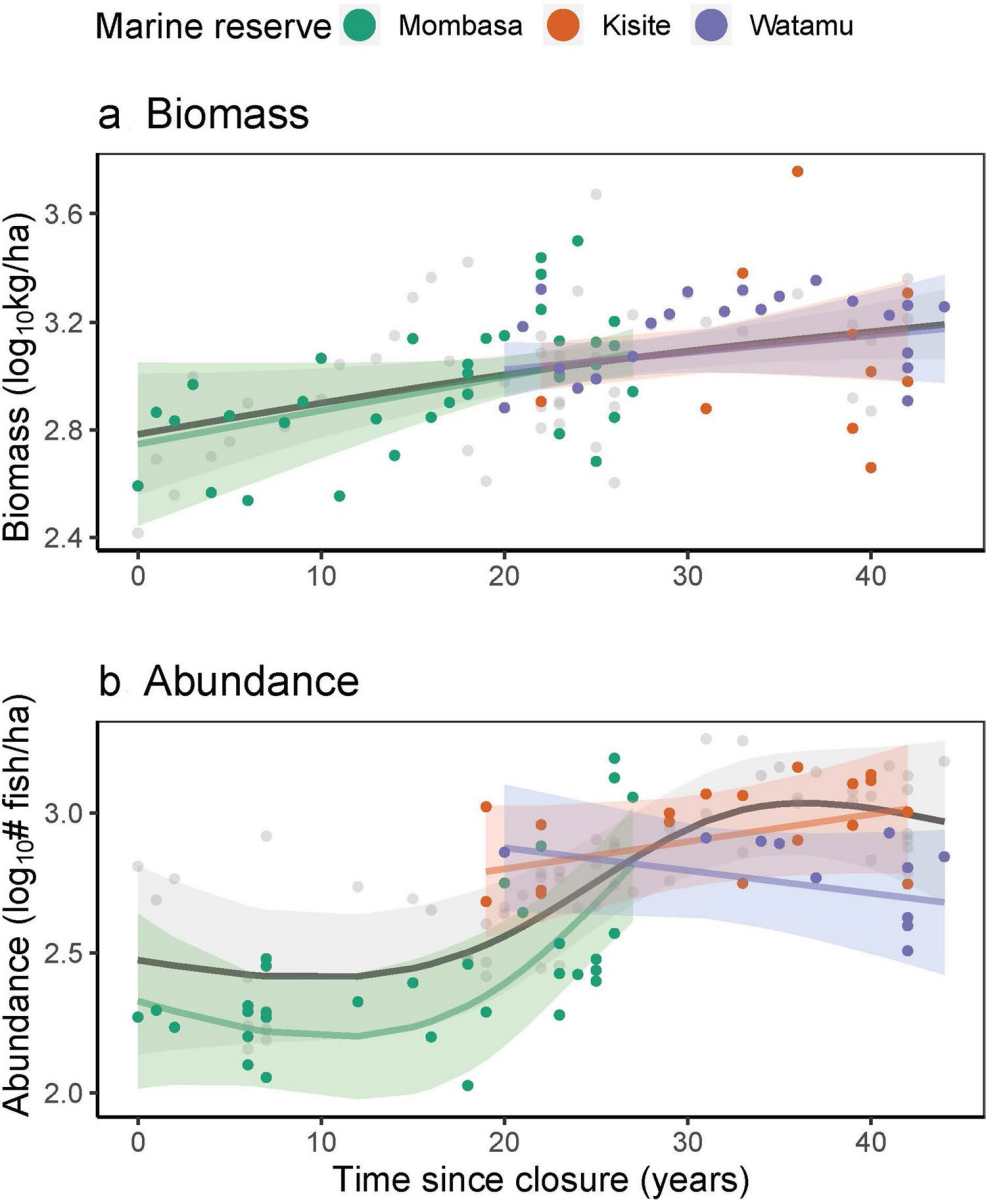
Modelled changes in (**a**) mean biomass (logged) and (**b**) mean abundance (logged) over time since closure of the marine parks, holding other covariates to their means, with 95% confidence intervals shaded. Points are partial residuals for the models with colours corresponding to the marine reserve, where Mombasa = green, Kisite = orange and Watamu = purple. The model with marine reserve as a random effect is illustrated in grey.

Biomass and abundance both increased over time since the cessation of fishing, while holding other covariates to their means (Fig. 1). However, while the slope of biomass was close to log-linear (EDF = 1.330, R^2^ = 0.255), with the rate of increase slowing just after 20 years of closure, the abundance curve was relatively flat to 17 years, and then steeply increased to a peak at 35 years (EDF = 2.68, R^2^ = 0.83; Supplementary Table S3). When the slope of the biomass curve was allowed to vary by marine reserve, the marine reserve trends remained very similar to the global trend. However, when the slope of the abundance curve was allowed to vary by marine reserve, only Mombasa had a significant, positive trend (EDF = 2.28, R^2^ = 0.74, *p* < 0.001). Kisite’s abundance slope was not significant when looked at independently (*p* = 0.20), but in the hierarchical global model, it appears to drive the steep increase in the overall trend, whereas, Watamu flattens the curve (Supplementary Table S3). Importantly, the overlap between abundance values in Kisite and Mombasa around 20 years of closure, indicates that this increase was more likely due to time since closure, rather than Kisite having a higher abundance of fish than Mombasa. There was a mass bleaching event in 1998, which corresponded to 7 years of closure for Mombasa, 25 years of closure for Kisite, and 26 years of closure for Watamu, but this does not appear to have an overall effect on the recovery trajectory of fish abundances in each of the reserves included in this analysis (Fig. 1b.; Supplementary Fig. S1).

### Functional space

**Figure 2:**
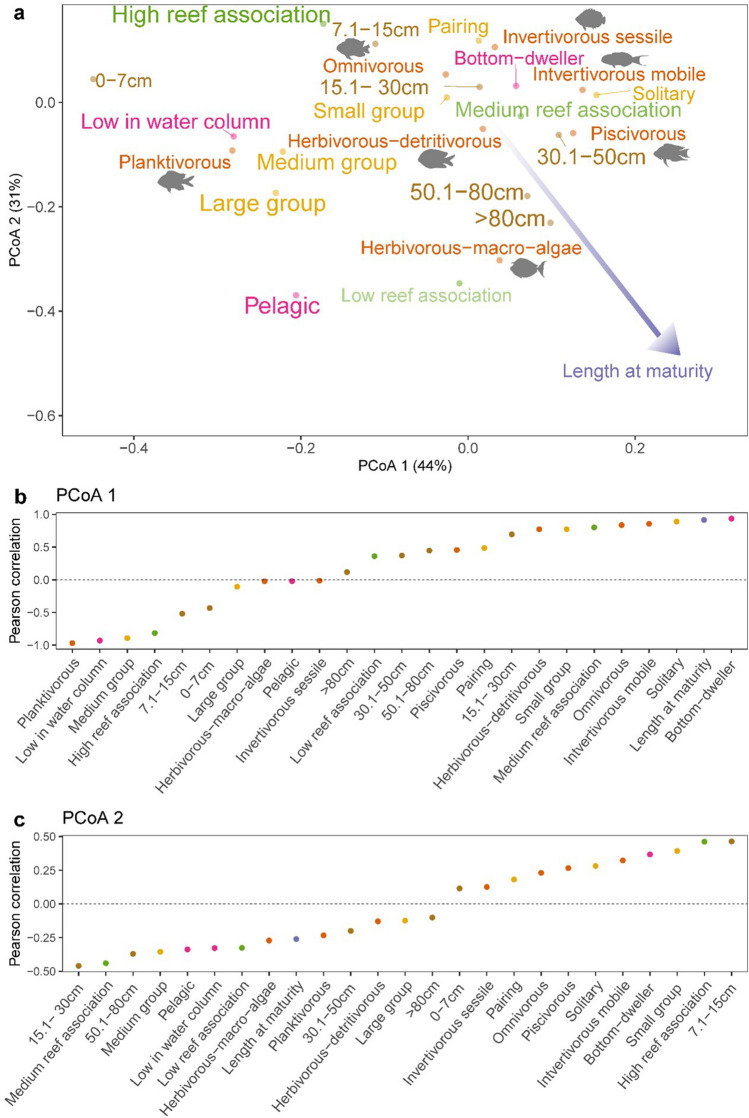
(**a**) Functional space of Kenyan marine parks across all sites and years spanning the chronosequence. Traits included: size, diet, gregariousness, position in the water column, reef association, length at maturity. Traits grouped by colour: purple = length at maturity, green = reef association, yellow = gregariousness, pink = position in the water column, brown = size, and orange = diet. A colour and size gradient are applied to each ordinal trait, increasing in size and opacity along the gradient. (**b**) Pearson correlation between community weighted mean values of trait categories and PCoA axis 1 and c) PCoA axis 2.

The first two PCoA axes captured 75% of the variation in the trait space of the 216 species assessed in this analysis (Fig. 2a). The top five trait categories most positively associated with axis 1 of the fish community PCoA are bottom-dweller, large length at maturity, solitary, invertivorous (mobile invertebrate feeders), and medium reef association. The most negatively associated traits with PCoA axis 1 were planktivorous, low in the water column, medium group, high reef association, and 7.1–15 cm sized fish (Fig. 2b). The top five traits most positively associated with PCoA axis 2 were 7.1–15 cm sized fish, high reef association, small group forming, bottom dweller, and invertivorous (mobile invertebrate feeders). The most negatively associated traits with PCoA axis 2 were 15.1–30 cm, medium reef association, 50.1–80 cm, medium group forming, and pelagic (Fig. 2c).

**Figure 3:**
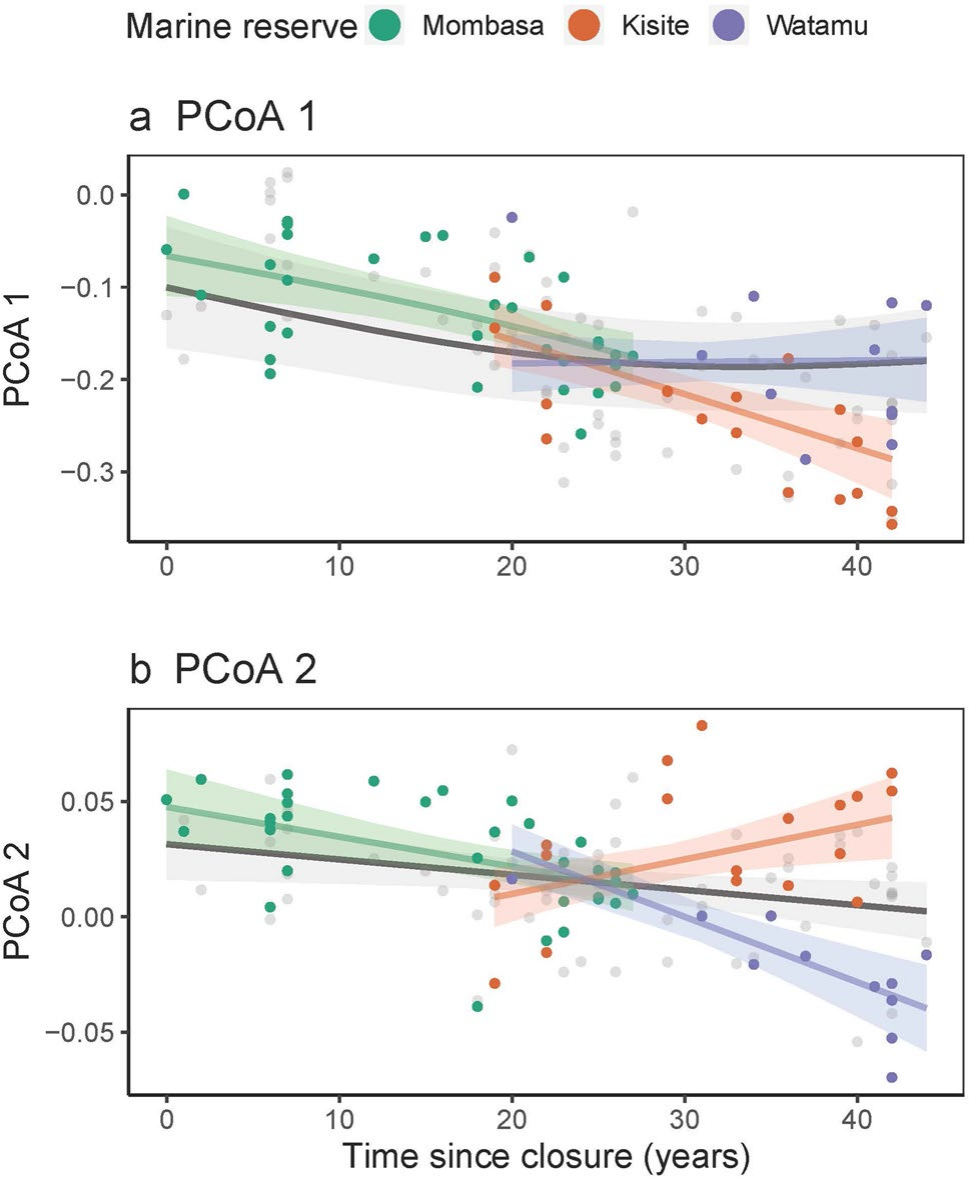
Modelled changes in (**a**) PCoA 1 and (**b**) PCoA 2 over time since closure of the marine parks, holding other covariates to their means, with 95% confidence intervals shaded. Points are partial residuals for the models with colours corresponding to the marine reserve, where Mombasa = green, Kisite = orange and Watamu = purple. The model with marine reserve as a random effect is illustrated in grey.

Both PCoA axes’ 1 and 2 mean community values had a negative relationship with time since closure of the marine parks, while holding other covariates to their means (Fig. 3), and the time smoother was significant for both axes (Axis 1, *p* = 0.01; Axis 2, *p* = 0.03). However, a greater proportion of the variance was described in the model by PCoA 1 (R^2^ = 0.75) compared to PCoA 2 (R^2^ = 0.44) (Supplementary Table S3). This indicated a shift from solitary bottom dwellers, with large lengths at maturity, and invertivorous diets, towards medium size group forming, high to medium level of reef association fish found low in the water column, sized 7–15 cm, with planktivorous diets. These traits were mostly represented by species in Pomacentridae, with *Chromis dimidiata, Chromis viridis, Neopomacentrus azysron*, and *Pomacentrus caeruleus* largely driving the trends (Supplementary Fig. S3). While the overall axis trends decreased, when the slopes were allowed to vary by marine park, we see a difference in trends between Kisite and Watamu. The PCoA axis 1 values for Kisite decreased significantly over time (*p* < 0.001), while the PCoA axis 1 values for Watamu do not change significantly over time (*p* = 0.908). For PCoA axis 2, Kisite increased over time (*p* = 0.012), while Watamu decreased over time (*p* < 0.001). However, the PCoA axis 1 model explained more variance (R^2^ = 0.645; deviance explained = 67%) than the PCoA axis 2 model (R^2^ = 0.48; deviance explained = 50.2%).

### Shifts in trait proportions and means

**Figure 4:**
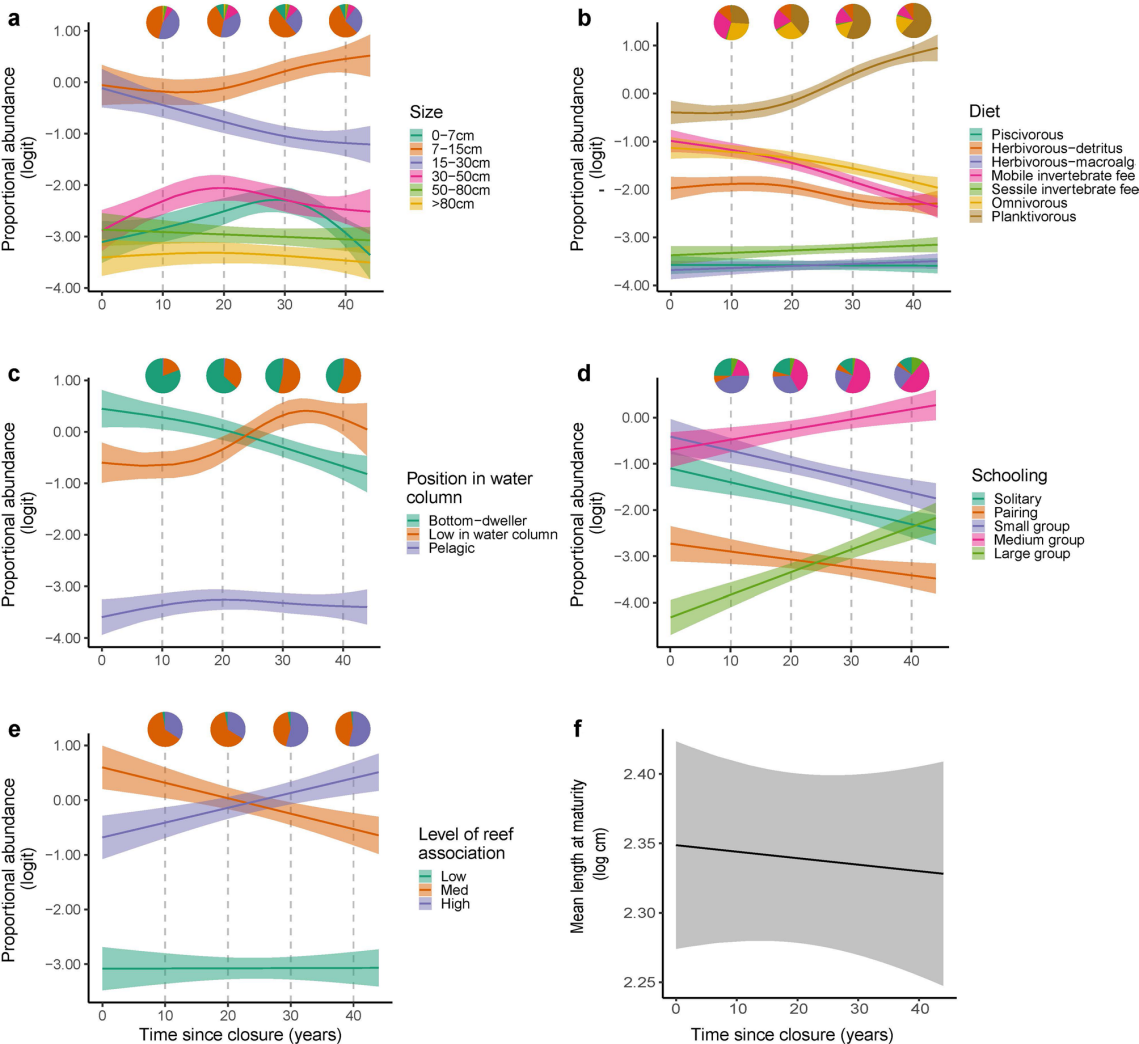
Modelled changes in proportional abundance of trait categories (**a**–**e**; (**a**) Size, (**b**) Diet, (**c**) Position in the water column, (**d**) Schooling, (**e**) Level of reef association) and mean values (**f** Mean length at maturity) of coral reef fish traits over a chronosequence of time since closure of marine parks, holding other covariates to their means, with 95% confidence intervals. Colours of the curves indicate the trait categories. Vertical dashed lines indicate 10 year marks in the chronosequence for which average trait category proportional abundances are illustrated in pie charts.

Individual trait proportions enable a clearer understanding of the mechanisms behind shifts in the multivariate trait space. We found that the majority of trait categories exhibited some change over time with protection (Fig. 4). Within the first 20 years of protection, a significant shift towards the increasing dominance of fish in the size-class 7–15 cm is observable, particularly increasing after 17 years, likely driving the overall abundance trend. The 15–30 cm size-class declined over time, while there was a slight increase in the proportion of fish in the 30–50 cm size-class between the beginning and end of the chronosequence, likely driving the overall biomass trend (Fig. 4a).

Planktivores, the most dominant diet category, become more proportionally abundant over time with protection (Fig. 4b). When holding all other model covariates to their means, the rate of increase in proportional abundance steepens after 20 years of protection and declines again after 30 years of protection (EDF = 2.60). Sessile invertebrate feeders, piscivores, and macroalgal feeders also increased, while detritivores, omnivores, and mobile invertebrate feeders decreased (Fig. 4b).

The proportion of pelagic fish recorded in the survey sites within the marine parks was consistently lower than both bottom-dwellers and fish low in the water column, likely due to the location of the survey sites on lagoonal back reefs. However, an increase in the dominance of fish low in the water column over bottom dwellers is observable after 20 years of protection, which corresponds to the first recordings of Watamu and Kisite marine reserves in the chronosequence (Fig. 4c). The random effect term “marine reserve” however, was not significant in the model, suggesting the patterns were more likely attributable to time since closure across the chronosequence (Supplementary Table S4).

Medium group-forming species, initially equally as dominant as solitary and small group (3–20 individuals) forming species become more dominant over time. All trends for schooling categories were linear or close to linear (EDF between 1.000031 and 1.000505). While large (> 50 individuals) groups increase over time, solitary, pairing, and small group (3–20 individuals) forming fish species decrease (Fig. 4d).

Patterns of change observed in levels of reef association were similar to those found for position in the water column. Fish with low levels of reef association were proportionally less abundant in the surveys than those with medium and high association across the time series, due to similar issues with sampling design that resulted in few pelagic fish being detected; Fig. 2a highlights the proximity of these two traits within the functional space. A switch from the dominance of medium to high levels of reef association can be observed after 20 years of protection (Fig. 4e).

The last trait assessed was an abundance-weighted mean of the continuous measure, length at maturity. Mean length at maturity did not significantly change over time (Fig. 4f), but this was likely due to the retrospective allocation of lengths at maturity at the species level, as intraspecific data on this were not available over time (see model outputs in Supplementary Table S5).

## Discussion

Developing our understanding of the mechanisms by which marine reserves affect ecosystem functioning is critical to identifying how, when, and if marine ecosystems recover from fishing^54^. We illustrate a shift in functional space over time with protection towards communities numerically dominated by fish in the size-class 7–15 cm, with a planktivorous diet, found low in the water column, forming medium-large schools, and with a high level of reef association. These findings were based on species’ trait abundances, and while both overall biomass and abundance increased over time, their patterns of increase differed.

The difference in shape between the biomass and abundance curves reflected community shifts occurring at the level of species’ traits. While the slope of the biomass curve increased steeply immediately following protection, the abundance curve did not follow suit until nearly 20 years of closure, when the rate of increase in biomass began to decline. The number and size of larger fish (e.g. 30–50 cm) increased early in the chronosequence, while the abundance of small, more proportionally abundant fish (e.g. 7–15 cm) did not increase significantly until 20 years of closure. This shift appeared to be largely driven by Kisite, which did not have as much absolute change in hard coral cover following the 1998 bleaching event as Watamu and Mombasa^55^. Kisite’s benthic PCA had a positive relationship with axis 2 of the fish community functional space, for which the 7–15 cm size class trait was strongly correlated (Supplementary Fig. S10). This was reflected in Kisite’s deviation from the overall trend in PCoA 2. Kisite marine reserve is located further offshore than the other two reserves, had less coral cover than the other reserves prior to 1998, and has less market gravity than both Mombasa and Watamu^56^. It is possible that these factors interacted to create a greater buffer against fish community change driven by disturbance to the benthos. After time since closure, thermal stress and benthic composition explained the most variance in the functional space models (Supplementary Fig. S7).

Because the fish trait size bins were somewhat arbitrary, as size is a continuous trait, and the 7–15 cm and 15–30 cm categories were sequential, the patterns observed were not easily distinguishable from those driven by shifts in species composition, a consequence of using an interspecific trait-based approach^10^. However, a sensitivity analysis revealed that even when the most abundant species in the 7–15 cm size-class, *Chromis dimidiata*, was removed, the same trends persisted (Supplementary Fig. S4). Larger bodied fish were likely to be driving overall biomass trends, while small fish were likely to be driving the overall abundance trends and appeared to be responding in sequence and contrary to the ecological succession expectation that small fish will respond more rapidly than large fish^57^. Perhaps the deviation from expectation occurs because fish in the 30–50 cm size-class were disproportionately targeted in Kenyan fisheries^58^, and therefore, they increase rapidly when released from predation. Smaller fish, in contrast, respond to slower contextual changes in the food web.

We hypothesised that there would be a decrease in smaller size-classes and an increase in larger size-classes, as fishing exploitation has been shown to increase the steepness of the slopes of coral reef fish size spectra, due partly to the effects of predation release^59^. Increased predation in reserves may therefore be expected to drive a reduction in smaller size-classes and an increase in larger size-classes. However, previous research has demonstrated that piscivores are not disproportionately caught in Kenyan fisheries, and therefore they do not experience the rapid recovery following protection that might lead to a decrease in smaller fish (Fig. 4)^58^. In geographies where piscivores are a more prominent component of the fish community, these patterns may differ. Similar work evaluating shifts in the biomass of trophic groups indicated that the overall trophic level of fish within Kenyan marine parks was decreasing over time as slow-growing herbivores come to dominate the biomass^29^. It may be that these small to modest-size urban parks are not large enough to support the space requirements of large piscivores^60^. Therefore, the responses observed here may only be applicable to these types of modest-size closures of < 10 km^2^.

The four most economically valuable fish families in Kenya, including Lutjanidae (Snappers), Lethrinidae (Emperors), Siganidae (Rabbitfishes), and Serranidae (Groupers), were not included in the list of eight families surveyed for the full duration of the chronosequence. The species list for this study comprised of mid-value and bycatch families that are more common in the fisheries (e.g. Scarinae)^61^ and contribute most to fish biodiversity. They make up the bulk of the abundance and biomass. Thus, the functional importance of the trait shifts observed in this study should be interpreted through the lens of the mass-ratio hypothesis—whereby it is the more abundant traits or species that have the greatest functional impact^62^. For example, for diet, the most abundant trait class (planktivores) became even more abundant with protection. Where the abundance of mainly small planktivores adds up to produce large proportions of the biomass, systems can be said to be “middle-driven”. These middle-driven trophic pyramids have been found to exist at high levels of biomass, regardless of protection regime^63,64^. Planktivores provide important pelagic subsidies to a reef, increasing overall productivity and playing a key role in nutrient cycling^65^. Many planktivores are also dependent on reef structure for recruitment and predator avoidance^66,67^. Their abundances have been shown to decline with coral bleaching and the loss of structural complexity and increase with protection from fishing^68,69^. Some planktivorous families, such as the Pomacentridae, are considered “bycatch” in Kenyan fisheries and are not specifically targeted. The increase in the proportional abundance of planktivores could therefore primarily be linked to the recovering habitat within protected areas^70,71^.

Evolutionarily, shifts to planktivory are linked to increasing schooling behaviour^72^. Our analysis showed that these trait categories, which tend to cluster, were both increasing over time with protection. An increase in the abundance of fish exhibiting gregarious behaviour has implications for functional processes related to how much fish consume. For example, Michael et al.^73^ found that both herbivory rates and the amount of algae consumed by three studied species were higher when individuals fed in monospecific groups. Social aggregations should theoretically lead to more protection, and therefore the increased ability to forage^74^. However, resource competition among those in the group can also lead to less overall consumption. It has been demonstrated that for a planktivorous species, this trade-off between protection and competition is mediated by the availability of resources^75^.

Competition within groups also affects life history characteristics dependent on environmental stochasticity, so that individuals in larger groups tend to have slower growth rates^76^. Interestingly, however, we did not see a significant positive response to protection in the length at maturity trait. This may be due to the interspecific approach taken in the analysis that doesn’t account for changes in the phenotypic plasticity of individuals and evolutionary adaptations inherited in specific populations over time^77,78^. Again, the patterns here may also be a function of the limited space of the closures that could exclude long-lived and late-reproducing species. These closures should not be viewed as undisturbed systems but rather islands within fished seascapes^79^. Nevertheless, given the interspecific approach, we would expect that considering the overall PCoA abundance trends towards smaller or moderate-sized species, these species would have smaller lengths at maturity. This is because length at maturity, like many traits, is highly correlated with size^80^.

The trait-based analyses presented in this paper were abundance-weighted. This provides a species-level approach to compliment previous family-level studies weighted by biomass investigating the Kenyan marine park system^29,81^. If intraspecific or species-level body sizes were available to evaluate biomass-weighted trends, it is possible that different patterns could emerge, with implications for ecosystem functioning. For example, families such as Labridae (Scarinae) and Acanthuridae have been shown to dominate the biomass of marine reserves in Kenya over time with protection from fishing^28^. These families consist of herbivorous and large-bodied fish, and their functional impact has been demonstrated in experiments^82^. Abundance-based metrics may not reflect the dominance of these groups as much as biomass-based metrics. Therefore, it is necessary to interpret these results as a component of a multi-faceted approach to understanding ecosystem processes as a function of both abundance and biomass. Furthermore, directly measuring ecosystem processes (e.g. herbivory, predation, etc.) would also provide a fuller picture^9^.

Increases in fish biomass, abundance, and the proportion of functionally important traits over time with high compliance protection is expected to represent recovery from fishing pressure^83,84^. However, this recovery is taking place in the context of a changing climate and a dynamic ocean^26,85^. While we see an increase in the biomass and abundance of fish in Kenya’s marine reserves, species and traits have not necessarily recovered, in that they have not returned to historic compositional “baselines”^86^. Incorporating the concept of a novel ecosystem into conservation moves away from the de facto goal of restoration to ecological baselines^17^. In this paper, we demonstrate how fish traits respond over time to the establishment of marine reserves. The resulting community after 44 years of protection appears to still be changing and not approaching a plateau. While some traits have become more dominant over time (e.g., 7–15 cm, planktivores), other traits (e.g., high gregariousness, high reef association) have started to surpass those that were previously dominant. This highlights the importance of interpreting patterns within the context in which marine reserves are situated, the dynamic nature of recovery, and the potential for novel trait configurations to shape the provision of altered ecosystem functions and services^87^.

## Supplementary Information

Supplementary Information.

## Data Availability

The data and code used for this study will be made available on https://github.com/Jeneen/trait_time_series.
