# Supplementary material for: Decadal shifts in traits of reef fish communities in marine reserves
Source: Sci Rep. 2021 Dec 6;11:23470. doi: 10.1038/s41598-021-03038-9 (PMC8648868; doi:10.1038/s41598-021-03038-9)
Supplement: Supplementary file 1 — Supplementary Information. [file 41598_2021_3038_MOESM1_ESM.docx]

| **Supplementary Table S1. Coral reef fish traits and justification for selection** | | | | |  |
| --- | --- | --- | --- | --- | --- |
| ***Trait*** | ***Trait grouping*** | ***Trait type*** | ***Source*** | ***Justification*** | |
| Total body length | Morphological | Ordinal: 0–7 cm < 7.1–15 cm < 15.1–30 cm < 30.1–50 cm < 50.1–80 cm < >80 cm | GASPAR | Size is known to respond to fishing pressure ^1^, and size-selective fishing tends to select for larger-bodied species ^2^. Size can determine the functional impact of a fish on processes such as nutrient cycling ^3^, bioerosion ^4^, herbivory ^5,6^, and predation ^7^. It corelates with a number of other traits that are not as easily measured but are known to have functional significance ^8^. | |
| Diet | Diet/trophic | Categorical: herbivorous-detritivorous (i.e., fish feeding on turf or filamentous algae and/or undefined organic material), macroalgal herbivorous (i.e., fish eating large fleshy algae and/or seagrass), invertivorous targeting sessile invertebrates (i.e., corals, sponges, ascidians), invertivorous targeting mobile invertebrate (i.e., benthic species such as crustaceans), planktivorous (i.e., fish eating small organisms in the water column), piscivorous (including fish and cephalopods), and omnivorous (i.e., fish for which both vegetal and animal material are important in their diet) | GASPAR | Higher trophic levels of fish tend to be more targeted by fishing and benefit more from protection ^9^. The diet of a fish influences which ecosystem processes species have an effect on by defining an essential axis of their ecological niche; for example, species can be predators that mediate the process of predation ^10^, herbivores that feed on algae and mediate competition with other benthic organisms ^11^, or bioeroders enabling sediment production and coral recovery ^12,13^. | |
| Schooling | Behaviour | Ordinal: solitary < pairing, or living in small (3–20 individuals) < medium (20–50 individuals) < large (>50 individuals) groups | GASPAR | The number of fish in a school has been shown to affect feeding and influence the total number of bites taken by fish ^14^. Schooling behaviour also influences predator-prey functional dynamics in a number of ways ^15^. Gregariousness is also known to interact with fishing. For example, schooling influences catachability of a species as well as how targeted a fish might be by certain fishing technologies ^16,17^. | |
| Position in the water column | Behaviour | Ordinal: bottom-dweller < bentho-pelagic (low in water column) < pelagic | GASPAR | Water column position influences potential prey items for predators, likelihood of predation for prey, and general resource use ^18,19^. Evolutionary shifts in water column position are linked to changes in body morphology ^20,21^. | |
| Reef association | Behaviour | Ordinal: low reef association < medium reef association < high reef association | Fishbase and expert opinion | Fishing pressure and protection from fishing can impact the benthic environment of coral reef ecosystems ^22,23^. Species that are dependent on the coral reef habitat are therefore vulnerable to the effects of fishing ^24^. While small fish, associated with high levels of reef association ^25^, are typically not targeted by fisheries in Kenya ^26^, fishing can impact the level of reef association of fish communities through indirect effects by damaging the benthos. The level of reef association of a fish will also determine where in space it is able to live and therefore have a functional impact ^27^. | |
| Length at maturity | Life history | Continuous | FishLife ^28^ | Life history traits, often grouped together into life history strategies, tend to be highly correlated ^29^. Length at maturity was selected as a representative life history trait because length-based life history measurements have been shown to be particularly sensitive to fishing pressure ^30,31^. Fishing can often have a greater negative impact on the abundances of long-lived, slow growing, late maturing, larger species, leading to changes in community compositions ^32,33^. | |

| **Supplementary Table S2. Final set of covariates included in the models and their justifications for use** | | |
| --- | --- | --- |
| ***Covariates*** | ***Justification*** | ***Calculation/source*** |
| Benthic PCA | The interplay between the coral reef habitat and the fish (abundance, biomass, and traits) that live in the habitat is complex^34^. We attempted to partially account for this by including a multivariate measure of the benthic habitat in the models. After some preliminary analyses, we decided to use this multivariate measure to address our questions, instead of looking at coral reef genus traits, because this measure sufficiently accounted for the benthic change over time at the scale we required. | A Principal Component Analysis (PCA) was conducted on percentage cover of 1) hard coral, 2) macroalgae, 3) coralline algae, and 4) other calcareous algae across all sites. This produced a succinct multivariate value (PCA axis 1 explaining 50% of the variation) for each site/year that captures multiple aspects of the benthos and at the same time reduced the number of parameters needed to be included in the models. |
| Rugosity | Rugosity, a measure of the structural complexity of the reef, was included as covariate in the global models separate to the PCA of the benthic community because it has been found to be an important predictor of reef fish abundance, biomass, and trophic structure ^34^. | Rugosity was measured in the field. |
| Thermal stress anomaly - lagged | Thermal Stress Anomalies (TSAs) were included in the models as they can potentially affect reef fish traits via two pathways. Firstly, they are associated with coral bleaching events, and therefore can alter the reefs that the fish depend on through a lagged effect. Secondly, McClanahan^35^ showed that variation in TSA is associated with the biomass of certain fish families; this could be through a benthic pathway or another unknown/unmeasured pathway. Fish communities are expected to exhibit a lagged response to disturbances such as thermal stress ^36^. | TSA data from 1991-2018 for each marine park were extracted from The Coral Reef Temperature Anomaly Database (CoRTAD) hosted by NOAA Coral Reef Watch. TSAs are calculated for 4 km grid cells as the weekly sea surface temperature minus the maximum weekly climatological (long-term average) sea surface temperature 34. The maximum TSA (magnitude) for each reserve in each year was selected for modelling. Therefore, the optimal time-lag for the effect of TSAs on fish functional space was assessed by lagging TSA values from 0 to 9 years and incorporating this lag into a GAMM model of the first PCoA axis. Lagged models were compared (for the same dataset years), and an optimal-fit lag of 4 years was selected to be included in the models, using the AIC selection procedure |
| Chlorophyll a | Chlorophyll a, a measure of phytoplankton density and therefore oceanic productivity, was included in the models because it is likely to influence reef fish trophic structure^37^ and the state of the coral reef habitat ^38^. | Oceanic productivity was estimated using chlorophyll a for the years 1997-2018, which were available from the Ocean Colour Climate Change Initiative dataset esa-cci-chla-monthly-v4-1 by the European Space Agency (http://www.esa‐oceancolour‐cci.org/). Daily data were averaged to get annual values at a 4-km resolution. For years prior to 1997, the average value of chlorophyll a for each park over the time-series was taken. |

| **Supplementary Table S3.a. Model output summaries (biomass, abundance, PCoA)** | | | | | | | | | | |  |  |  |  |  |  |
| --- | --- | --- | --- | --- | --- | --- | --- | --- | --- | --- | --- | --- | --- | --- | --- | --- |
|  | **Biomass (log 10 kg/ha)** | | | | **Abundance (log 10 fish/ha)** | | | | **PCoA axis 1** | | | | **PCoA axis 2** | | | |
| ***Optimal model*** | *biomass ~ s(time) + re(MPA) + VarPower (~time) + corAR1(~year \| site)* | | | | *abundance ~ s(time) + s(chlorophyll a) + s(tsa_lagged) + re(MPA) + corAR1(~year \| site)* | | | | *PCoA axis 1 ~ s(time) + s(tsa_lagged) + s(rugosity) + re(MPA) + corAR1(~year \| site)* | | | | *PCoA axis 2 ~ s(time) + s(tsa_lagged) + s(rugosity) + s(benthic PCA 1) + re(MPA) + corAR1(~year \| site)* | | | |
| ***Predictors*** | ***Estimates*** | ***CI*** | ***p*** | ***EDF*** | ***Estimates*** | ***CI*** | ***p*** | ***EDF*** | ***Estimates*** | ***CI*** | ***p*** | ***EDF*** | ***Estimates*** | ***CI*** | ***p*** | ***EDF*** |
| (Intercept) | 3.04 | 2.98 – 3.10 | **<0.001** |  | 2.80 | 2.76 – 2.84 | **<0.001** |  | -0.16 | -0.18 – -0.15 | **<0.001** |  | 0.02 | 0.01 – 0.03 | **<0.001** |  |
| Smooth term (Time since closure) |  |  | **0.012** | 1.330449 |  |  | **<0.001** | 2.678 |  |  | **0.01** | 1.919623 |  |  | **0.026** | 1.000453 |
| Smooth term, random effect (MPA) |  |  | 0.965 | 5.41E-09 |  |  | 0.820 | 1.01E-06 |  |  | 0.178 | 0.495737 |  |  | 0.6 | 1.59E-05 |
| Smooth term (Chlorophyll a) |  |  |  |  |  |  | **<0.001** | 4.704 |  |  | **<0.001** | 3.945773 |  |  |  |  |
| Smooth term (TSA max lagged 4 years) |  |  |  |  |  |  | **<0.001** | 1 |  |  | **<0.001** | 2.436247 |  |  |  |  |
| Smooth term (Rugosity) |  |  |  |  |  |  |  |  |  |  | **0.016** | 1.000088 |  |  | **0.004** | 1.000032 |
| Smooth term (benthic PCA 1) |  |  |  |  |  |  | 0.017 | 5.288 |  |  |  |  |  |  | **<0.001** | 1.000058 |
| Observations | 69 | | | | 61 | | | | 61 | | | | 61 | | | |
| R^2^ | 0.255 | | | | 0.827 | | | | 0.751 | | | | 0.439 | | | |
| Deviance explained |  | | | | 86.6% | | | | 79.10% | | | | 46.70% | | | |

| **Supplementary table S3.b. Model output summaries (biomass, abundance, PCoA by marine reserve)** | | | | | | | | | | | | | |  |  |  |
| --- | --- | --- | --- | --- | --- | --- | --- | --- | --- | --- | --- | --- | --- | --- | --- | --- |
|  | **Biomass (log 10 kg/ha)** | | | | **Abundance (log 10 fish/ha)** | | | | **PCoA axis 1** | | | | **PCoA axis 2** | | | |
| ***Optimal model*** | *biomass ~ s(time, k = 4, bs = "cr",   by = MPA)* | | | | *abundance ~ s(time, k = 4, bs = "cr",   by = MPA) + s(chlorophyll a, bs = "cr") + s(tsa lagged,   bs = "cr")* | | | | *PCoA axis 1 ~ s(time, k = 4, by = MPA, bs = "cr") +   s(tsa lagged, bs = "cr")* | | | | *PCoA axis 2 ~ s(time, k = 4, by = MPA, bs = "cr")* | | | |
| ***Predictors*** | ***Estimates*** | ***CI*** | ***p*** | ***EDF*** | ***Estimates*** | ***CI*** | ***p*** | ***EDF*** | ***Estimates*** | ***CI*** | ***p*** | ***EDF*** | ***Estimates*** | ***CI*** | ***p*** | ***EDF*** |
| (Intercept) | 3.05 | 2.98-3.31 | **<0.001** |  | 2.968 | 2.80 – 3.14 | **<0.001** |  | -0.17 | -0.20 – -0.15 | **<0.001** |  | 0.02 | 0.01 – 0.022 | **0.001** |  |
| Smooth term (Time since closure) *Mombasa |  |  | 0.0685 | 1 |  |  | **0.001** | 2.283 |  |  | **0.001** | 1.276 |  |  | **0.003** | 1 |
| Smooth term (Time since closure) *Kisite |  |  | 0.2759 | 1 |  |  | 0.197 | 1 |  |  | **<0.001** | 1 |  |  | **0.012** | 1 |
| Smooth term (Time since closure) *Watamu |  |  | 0.2695 | 1 |  |  | 0.291 | 1 |  |  | **0.908** | 1 |  |  | **<0.001** | 1 |
| Smooth term (Chlorophyll a) |  |  |  |  |  |  | **<0.001**  5.181 | |  |  |  |  |  |  |  |  |
| Smooth term (TSA max lagged 4 years) |  |  |  |  |  |  | **<0.001** 1 | |  |  | **<0.001** | 1 |  |  |  |  |
| Smooth term (Rugosity) |  |  |  |  |  |  |  |  |  |  |  |  |  |  |  |  |
| Smooth term (benthic PCA 1) |  |  |  |  |  |  |  |  |  |  |  |  |  |  |  |  |
| Observations | 69 | | | | 61 | | | | 61 | | | | 61 | | | |
| R^2^ | 0.227 | | | | 0.743 | | | | 0.645 | | | | 0.476 | | | |
| Deviance explained |  | | | |  | | | | 67.00% | | | | 50.20% | | | |

| **Supplementary Table S4. Model output summaries (individual traits - categorical)** | | | | | | | | | | | | | | | | | | |  |  |
| --- | --- | --- | --- | --- | --- | --- | --- | --- | --- | --- | --- | --- | --- | --- | --- | --- | --- | --- | --- | --- |
|  | **Size (logit)** | | | | **Diet (logit)** | | | | **Position in water column (logit)** | | | | **Schooling (logit)** | | | | **Reef association (logit)** | | | |
| ***Optimal model*** | *proportional abundance of trait category ~ s(time by size category) + size + re(MPA) + corAR1(~year \| site)* | | | | *proportional abundance of trait category ~ s(time by diet category) + diet + s(tsa_lagged) + re(MPA) + corAR1(~year \| site)* | | | | *proportional abundance of trait category ~ s(time by position category) + position + s(rugosity) + re(MPA) + corAR1(~year \| site)* | | | | *proportional abundance of trait category ~ s(time by schooling category) + schooling + s(benthic PCA 1) + re(MPA) + corAR1(~year \| site)* | | | | *proportional abundance of trait category ~ s(time by association category) + association + re(MPA) + corAR1(~year \| site)* | | | |
| ***Predictors*** | ***Estimates*** | ***CI*** | ***p*** | ***EDF*** | ***Estimates*** | ***CI*** | ***p*** | ***EDF*** | ***Estimates*** | ***CI*** | ***p*** | ***EDF*** | ***Estimates*** | ***CI*** | ***p*** | ***EDF*** | ***Estimates*** | ***CI*** | ***p*** | ***EDF*** |
| (Intercept) | -2.7 | -2.86 – -2.55 | **<0.001** |  | -3.57 | -3.66 – -3.48 | **<0.001** |  | -0.16 | -0.31 – -0.01 | **0.032** |  | -1.83 | -2.01 – -1.65 | **<0.001** |  | -3.07 | -3.26 – -2.89 | **<0.001** |  |
| Smooth term (Time since closure) |  |  |  |  |  |  |  |  |  |  |  |  |  |  |  |  |  |  |  |  |
| Smooth term, random effect (MPA) | 0.6 |  | 0.206 | 0.597316 | 0.48 |  | 0.232 | 0.480526 | 0 |  | 0.862 | 1.31E-05 | 0 |  | 0.849 | 1.39E-05 | 0 |  | 1 | 0.000143 |
| Smooth term (Chlorophyll a) |  |  |  |  |  |  |  |  |  |  |  |  |  |  |  |  |  |  |  |  |
| Smooth term (TSA max lagged 4 years) |  |  |  |  | 1 |  | 0.063 |  |  |  |  |  |  |  |  |  |  |  |  |  |
| Smooth term (Rugosity) |  |  |  |  |  |  |  |  | 1 |  | 0.054 | 1.000068 |  |  |  |  |  |  |  |  |
| Smooth term (benthic PCA 1) |  |  |  |  |  |  |  |  |  |  |  |  | 1 |  | **0.043** | 1.000779 |  |  |  |  |
| Size2 (7.1-15cm) | 2.77 | 2.56 – 2.97 | **<0.001** |  |  |  |  |  |  |  |  |  |  |  |  |  |  |  |  |  |
| Size3 (15.1- 30cm) | 1.84 | 1.63 – 2.04 | **<0.001** |  |  |  |  |  |  |  |  |  |  |  |  |  |  |  |  |  |
| Size4 (30.1-50cm) | 0.36 | 0.15 – 0.56 | **0.001** |  |  |  |  |  |  |  |  |  |  |  |  |  |  |  |  |  |
| Size5 (50.1-80cm) | -0.31 | -0.51 – -0.10 | **0.003** |  |  |  |  |  |  |  |  |  |  |  |  |  |  |  |  |  |
| Size6 (>80cm) | -0.71 | -0.91 – -0.50 | **<0.001** |  |  |  |  |  |  |  |  |  |  |  |  |  |  |  |  |  |
| Smooth term (Time since closure) |  |  |  |  |  |  |  |  |  |  |  |  |  |  |  |  |  |  |  |  |
| x Size1 (0-7cm) | 2.71 |  | **0.001** | 2.712033 |  |  |  |  |  |  |  |  |  |  |  |  |  |  |  |  |
| x Size2 (7.1-15cm) | 2.22 |  | **0.008** | 2.217212 |  |  |  |  |  |  |  |  |  |  |  |  |  |  |  |  |
| x Size3 (15.1- 30cm) | 1.79 |  | **<0.001** | 1.790748 |  |  |  |  |  |  |  |  |  |  |  |  |  |  |  |  |
| x Size4 (30.1-50cm) | 2.48 |  | **0.009** | 2.476543 |  |  |  |  |  |  |  |  |  |  |  |  |  |  |  |  |
| x Size5 (50.1-80cm) | 1 |  | 0.404 | 1.000569 |  |  |  |  |  |  |  |  |  |  |  |  |  |  |  |  |
| x Size6 (>80cm) | 1.53 |  | 0.595 | 1.525904 |  |  |  |  |  |  |  |  |  |  |  |  |  |  |  |  |
| DietHD |  |  |  |  | 1.49 | 1.37 – 1.62 | **<0.001** |  |  |  |  |  |  |  |  |  |  |  |  |  |
| DietHM |  |  |  |  | 0 | -0.12 – 0.13 | 0.946 |  |  |  |  |  |  |  |  |  |  |  |  |  |
| DietIM |  |  |  |  | 1.93 | 1.81 – 2.05 | **<0.001** |  |  |  |  |  |  |  |  |  |  |  |  |  |
| DietIS |  |  |  |  | 0.33 | 0.21 – 0.45 | **<0.001** |  |  |  |  |  |  |  |  |  |  |  |  |  |
| DietOM |  |  |  |  | 2.1 | 1.97 – 2.22 | **<0.001** |  |  |  |  |  |  |  |  |  |  |  |  |  |
| DietPK |  |  |  |  | 3.74 | 3.62 – 3.87 | **<0.001** |  |  |  |  |  |  |  |  |  |  |  |  |  |
| Smooth term (Time since closure) |  |  |  |  |  |  |  |  |  |  |  |  |  |  |  |  |  |  |  |  |
| x DietFC |  |  |  |  | 1 |  | 0.901 | 1.00003 |  |  |  |  |  |  |  |  |  |  |  |  |
| x DietHD |  |  |  |  | 2.47 |  | **0.002** | 2.471613 |  |  |  |  |  |  |  |  |  |  |  |  |
| x DietHM |  |  |  |  | 1 |  | 0.215 | 1.00003 |  |  |  |  |  |  |  |  |  |  |  |  |
| x DietIM |  |  |  |  | 2 |  | **<0.001** | 2.002488 |  |  |  |  |  |  |  |  |  |  |  |  |
| x DietIS |  |  |  |  | 1 |  | 0.145 | 1.000046 |  |  |  |  |  |  |  |  |  |  |  |  |
| x DietOM |  |  |  |  | 1.83 |  | **<0.001** | 1.831862 |  |  |  |  |  |  |  |  |  |  |  |  |
| x DietPK |  |  |  |  | 2.6 |  | **<0.001** | 2.602779 |  |  |  |  |  |  |  |  |  |  |  |  |
| Position2 |  |  |  |  |  |  |  |  | 0.06 | -0.15 – 0.27 | 0.547 |  |  |  |  |  |  |  |  |  |
| Position3 |  |  |  |  |  |  |  |  | -3.2 | -3.41 – -3.00 | **<0.001** |  |  |  |  |  |  |  |  |  |
| Smooth term (Time since closure) |  |  |  |  |  |  |  |  |  |  |  |  |  |  |  |  |  |  |  |  |
| x Position1 (benthic) |  |  |  |  |  |  |  |  | 1.65 |  | **<0.001** | 1.646404 |  |  |  |  |  |  |  |  |
| x Position2 (low in water colum) |  |  |  |  |  |  |  |  | 2.74 |  | **<0.001** | 2.735291 |  |  |  |  |  |  |  |  |
| x Position3 (pelagic) |  |  |  |  |  |  |  |  | 1 |  | 0.227 | 1.000027 |  |  |  |  |  |  |  |  |
| Schooling2 (Pairing) |  |  |  |  |  |  |  |  |  |  |  |  | -1.31 | -1.57 – -1.06 | **<0.001** |  |  |  |  |  |
| Schooling3 (Small group) |  |  |  |  |  |  |  |  |  |  |  |  | 0.69 | 0.43 – 0.94 | **<0.001** |  |  |  |  |  |
| Schooling4 (Medium group) |  |  |  |  |  |  |  |  |  |  |  |  | 1.67 | 1.41 – 1.92 | **<0.001** |  |  |  |  |  |
| Schooling5 (Large group) |  |  |  |  |  |  |  |  |  |  |  |  | -1.3 | -1.56 – -1.05 | **<0.001** |  |  |  |  |  |
| Smooth term (Time since closure) |  |  |  |  |  |  |  |  |  |  |  |  |  |  |  |  |  |  |  |  |
| x Schooling1 (Solitary) |  |  |  |  |  |  |  |  |  |  |  |  | 1 |  | **<0.001** | 1.000128 |  |  |  |  |
| x Schooling2 (Pairing) |  |  |  |  |  |  |  |  |  |  |  |  | 1 |  | **0.015** | 1.000139 |  |  |  |  |
| x Schooling3 (Small group) |  |  |  |  |  |  |  |  |  |  |  |  | 1 |  | **<0.001** | 1.000031 |  |  |  |  |
| x Schooling4 (Medium group) |  |  |  |  |  |  |  |  |  |  |  |  | 1 |  | **0.002** | 1.000159 |  |  |  |  |
| x Schooling5 (Large group) |  |  |  |  |  |  |  |  |  |  |  |  | 1 |  | **<0.001** | 1.000505 |  |  |  |  |
| Reef.AssociationMed |  |  |  |  |  |  |  |  |  |  |  |  |  |  |  |  | 2.99 | 2.72 – 3.26 | **<0.001** |  |
| Reef.AssociationHigh |  |  |  |  |  |  |  |  |  |  |  |  |  |  |  |  | 3.05 | 2.78 – 3.32 | **<0.001** |  |
| Smooth term (Time since closure) |  |  |  |  |  |  |  |  |  |  |  |  |  |  |  |  |  |  |  |  |
| x Reef.AssociationLow |  |  |  |  |  |  |  |  |  |  |  |  |  |  |  |  | 1 |  | 0.964 | 1.000109 |
| x Reef.AssociationMed |  |  |  |  |  |  |  |  |  |  |  |  |  |  |  |  | 1 |  | **<0.001** | 1.000221 |
| x Reef.AssociationHigh |  |  |  |  |  |  |  |  |  |  |  |  |  |  |  |  | 1 |  | **<0.001** | 1.004593 |
| Observations | 366 | | | | 427 | | | | 173 | | | | 305 | | | | 183 | | | |
| R^2^ | 0.834 | | | | 0.935 | | | | 0.89 | | | | 0.747 | | | | 0.79 | | | |
| Deviance explained | 84.20% | | | | 93.80% | | | | 89.50% | | | | 75.50% | | | | 79.60% | | | |

| **Supplementary Table S5. Model output summaries (individual traits - continuous)** | | | | | |
| --- | --- | --- | --- | --- | --- |
|  | **Mean length at maturity (log cm)** | | | |  |
| ***Optimal model*** | *mean length ~ s(time) + s(tsa_lagged) + s(rugosity) s(benthic PCA 1) +s(chlorophyll a) + re(MPA) + corAR1(~year \| site)* | | | |  |
| ***Predictors*** | ***Estimates*** | ***CI*** | ***p*** | ***EDF*** |  |
| (Intercept) | 23.6 | 23.44 – 23.77 | **<0.001** |  |  |
| Smooth term (Time since closure) | 2.72 |  | 0.677 | 1.000017 |  |
| Smooth term, random effect (MPA) | 1 |  | 1 | 7.69E-06 |  |
| Smooth term (Chlorophyll a) | 39.08 |  | **0.001** | 3.665715 |  |
| Smooth term (TSA max lagged 4 years) | 2.72 |  | **<0.001** | 1.000525 |  |
| Smooth term (Rugosity) | 2.72 |  | **0.007** | 1.000015 |  |
| Smooth term (benthic PCA 1) | 63.51 |  | **0.018** | 4.151209 |  |
| Observations | 61 | | | |  |
| R^2^ | 0.708 | | | |  |
| Deviance explained | 75.80% | | | |  |

| **Supplementary Table S6. Species list** | | | | |
| --- | --- | --- | --- | --- |
| *Abudefduf septemfasciatus* | *Chaetodon flavirostris* | *Diodon eydouxii* | *Oxymonacanthus longirostris* | *Stegastes limbatus* |
| *Abudefduf sexfasciatus* | *Chaetodon guttatissimus* | *Diodon holocanthus* | *Paracanthurus hepatus* | *Stegastes lividus* |
| *Abudefduf sparoides* | *Chaetodon kleinii* | *Diodon hystrix* | *Paraluteres prionurus* | *Stegastes nigricans* |
| *Abudefduf vaigiensis* | *Chaetodon leucopleura* | *Diodon liturosus* | *Pervagor janthinosoma* | *Stegastes pelicieri* |
| *Acanthurus auranticavus* | *Chaetodon lineolatus* | *Epibulus insidiator* | *Pervagor melanocephalus* | *Stethojulis albovittata* |
| *Acanthurus blochii* | *Chaetodon lunula* | *Forcipiger flavissimus* | *Plectroglyphidodon dickii* | *Stethojulis interrupta* |
| *Acanthurus dussumieri* | *Chaetodon madagaskariensis* | *Forcipiger longirostris* | *Plectroglyphidodon johnstonianus* | *Stethojulis strigiventer* |
| *Acanthurus leucosternon* | *Chaetodon melannotus* | *Gomphosus caeruleus* | *Plectroglyphidodon lacrymatus* | *Sufflamen bursa* |
| *Acanthurus lineatus* | *Chaetodon meyeri* | *Halichoeres cosmetus* | *Pomacanthus chrysurus* | *Sufflamen chrysopterum* |
| *Acanthurus mata* | *Chaetodon trifascialis* | *Halichoeres hortulanus* | *Pomacanthus imperator* | *Sufflamen fraenatum* |
| *Acanthurus nigricauda* | *Chaetodon trifasciatus* | *Halichoeres iridis* | *Pomacanthus maculosus* | *Thalassoma amblycephalum* |
| *Acanthurus nigrofuscus* | *Chaetodon unimaculatus* | *Halichoeres lapillus* | *Pomacanthus rhomboides* | *Thalassoma genivittatum* |
| *Acanthurus nubilus* | *Chaetodon vagabundus* | *Halichoeres marginatus* | *Pomacanthus semicirculatus* | *Thalassoma hardwicke* |
| *Acanthurus tennentii* | *Chaetodon xanthocephalus* | *Halichoeres nebulosus* | *Pomacentrus agassizii* | *Thalassoma hebraicum* |
| *Acanthurus thompsoni* | *Chaetodon zanzibarensis* | *Halichoeres nigrescens* | *Pomacentrus baenschi* | *Thalassoma lunare* |
| *Acanthurus triostegus* | *Cheilinus fasciatus* | *Halichoeres scapularis* | *Pomacentrus caeruleus* | *Thalassoma lutescens* |
| *Acanthurus xanthopterus* | *Cheilinus oxycephalus* | *Hemigymnus fasciatus* | *Pomacentrus pavo* | *Thalassoma purpureum* |
| *Acreichthys radiatus* | *Cheilinus trilobatus* | *Hemigymnus melapterus* | *Pomacentrus pikei* | *Zebrasoma scopas* |
| *Acreichthys tomentosus* | *Cheilinus undulatus* | *Hemitaurichthys zoster* | *Pomacentrus sulfureus* | *Zebrasoma velifer* |
| *Aluterus monoceros* | *Cheilio inermis* | *Heniochus acuminatus* | *Pomacentrus trichrourus* |  |
| *Aluterus scriptus* | *Chlorurus atrilunula* | *Hipposcarus harid* | *Pomacentrus trilineatus* |  |
| *Amanses scopas* | *Chlorurus gibbus* | *Hologymnosus annulatus* | *Pomacentrus tripunctatus* |  |
| *Amblyglyphidodon leucogaster* | *Chlorurus sordidus* | *Hologymnosus doliatus* | *Pomachromis richardsoni* |  |
| *Amphiprion akallopisos* | *Chlorurus strongylocephalus* | *Labrichthys unilineatus* | *Pseudalutarius nasicornis* |  |
| *Amphiprion allardi* | *Chromis agilis* | *Labroides bicolor* | *Pseudobalistes flavimarginatus* |  |
| *Amphiprion latifasciatus* | *Chromis dimidiata* | *Labroides dimidiatus* | *Pseudobalistes fuscus* |  |
| *Anampses caeruleopunctatus* | *Chromis nigrura* | *Labropsis xanthonota* | *Pseudocheilinus evanidus* |  |
| *Anampses lineatus* | *Chromis opercularis* | *Leptoscarus vaigiensis* | *Pseudocheilinus hexataenia* |  |
| *Anampses meleagrides* | *Chromis pembae* | *Macropharyngodon bipartitus* | *Pseudodax moluccanus* |  |
| *Anampses twistii* | *Chromis ternatensis* | *Macropharyngodon cyanoguttatus* | *Pseudojuloides cerasinus* |  |
| *Apolemichthys trimaculatus* | *Chromis vanderbilti* | *Melichthys indicus* | *Pteragogus flagellifer* |  |
| *Balistapus undulatus* | *Chromis viridis* | *Melichthys niger* | *Pteragogus pelycus* |  |
| *Balistoides conspicillum* | *Chromis weberi* | *Naso annulatus* | *Pygoplites diacanthus* |  |
| *Balistoides viridescens* | *Chromis xutha* | *Naso brachycentron* | *Rhinecanthus aculeatus* |  |
| *Bodianus anthioides* | *Chrysiptera annulata* | *Naso brevirostris* | *Rhinecanthus rectangulus* |  |
| *Bodianus axillaris* | *Chrysiptera biocellata* | *Naso elegans* | *Scarus caudofasciatus* |  |
| *Bodianus bilunulatus* | *Chrysiptera brownriggii* | *Naso fageni* | *Scarus falcipinnis* |  |
| *Bodianus diana* | *Chrysiptera glauca* | *Naso francolina* | *Scarus ferrugineus* |  |
| *Calotomus carolinus* | *Chrysiptera unimaculata* | *Naso hexacanthus* | *Scarus festivus* |  |
| *Cantherhines dumerilii* | *Cirrhilabrus exquisitus* | *Naso tuberosus* | *Scarus frenatus* |  |
| *Cantherhines fronticinctus* | *Coris aygula* | *Naso unicornis* | *Scarus ghobban* |  |
| *Cantherhines pardalis* | *Coris caudimacula* | *Naso vlamingii* | *Scarus globiceps* |  |
| *Centropyge acanthops* | *Coris cuvieri* | *Neoglyphidodon melas* | *Scarus niger* |  |
| *Centropyge bispinosa* | *Coris formosa* | *Neopomacentrus azysron* | *Scarus psittacus* |  |
| *Centropyge multispinis* | *Ctenochaetus binotatus* | *Neopomacentrus cyanomos* | *Scarus rubroviolaceus* |  |
| *Cetoscarus bicolor* | *Ctenochaetus striatus* | *Novaculichthys taeniourus* | *Scarus russelii* |  |
| *Chaetodon auriga* | *Ctenochaetus strigosus* | *Odonus niger* | *Scarus scaber* |  |
| *Chaetodon bennetti* | *Dascyllus aruanus* | *Oxycheilinus bimaculatus* | *Scarus tricolor* |  |
| *Chaetodon blackburnii* | *Dascyllus carneus* | *Oxycheilinus digramma* | *Scarus viridifucatus* |  |
| *Chaetodon falcula* | *Dascyllus trimaculatus* | *Oxycheilinus mentalis* | *Stegastes fasciolatus* |  |

| **Supplementary Table S7. Sites and transects** | | | |
| --- | --- | --- | --- |
| **Reserve** | **Year** | **Number of transects** |  |
| Mombasa | 1991 | 9 |  |
| Kisite | 1992 | 5 |  |
| Mombasa | 1992 | 5 |  |
| Watamu | 1992 | 4 |  |
| Mombasa | 1993 | 5 |  |
| Kisite | 1995 | 7 |  |
| Mombasa | 1997 | 24 |  |
| Mombasa | 1998 | 12 |  |
| Kisite | 2002 | 4 |  |
| Mombasa | 2003 | 12 |  |
| Watamu | 2003 | 4 |  |
| Kisite | 2004 | 2 |  |
| Kisite | 2006 | 4 |  |
| Mombasa | 2006 | 4 |  |
| Watamu | 2006 | 4 |  |
| Mombasa | 2007 | 4 |  |
| Watamu | 2007 | 4 |  |
| Kisite | 2009 | 4 |  |
| Mombasa | 2009 | 8 |  |
| Watamu | 2009 | 2 |  |
| Mombasa | 2010 | 4 |  |
| Mombasa | 2011 | 2 |  |
| Kisite | 2012 | 4 |  |
| Mombasa | 2012 | 2 |  |
| Kisite | 2013 | 3 |  |
| Mombasa | 2013 | 2 |  |
| Watamu | 2013 | 2 |  |
| Mombasa | 2014 | 3 |  |
| Watamu | 2014 | 4 |  |
| Kisite | 2015 | 2 |  |
| Mombasa | 2015 | 2 |  |
| Mombasa | 2016 | 6 |  |
| Watamu | 2016 | 2 |  |
| Mombasa | 2017 | 3 |  |
| Mombasa | 2018 | 2 |  |


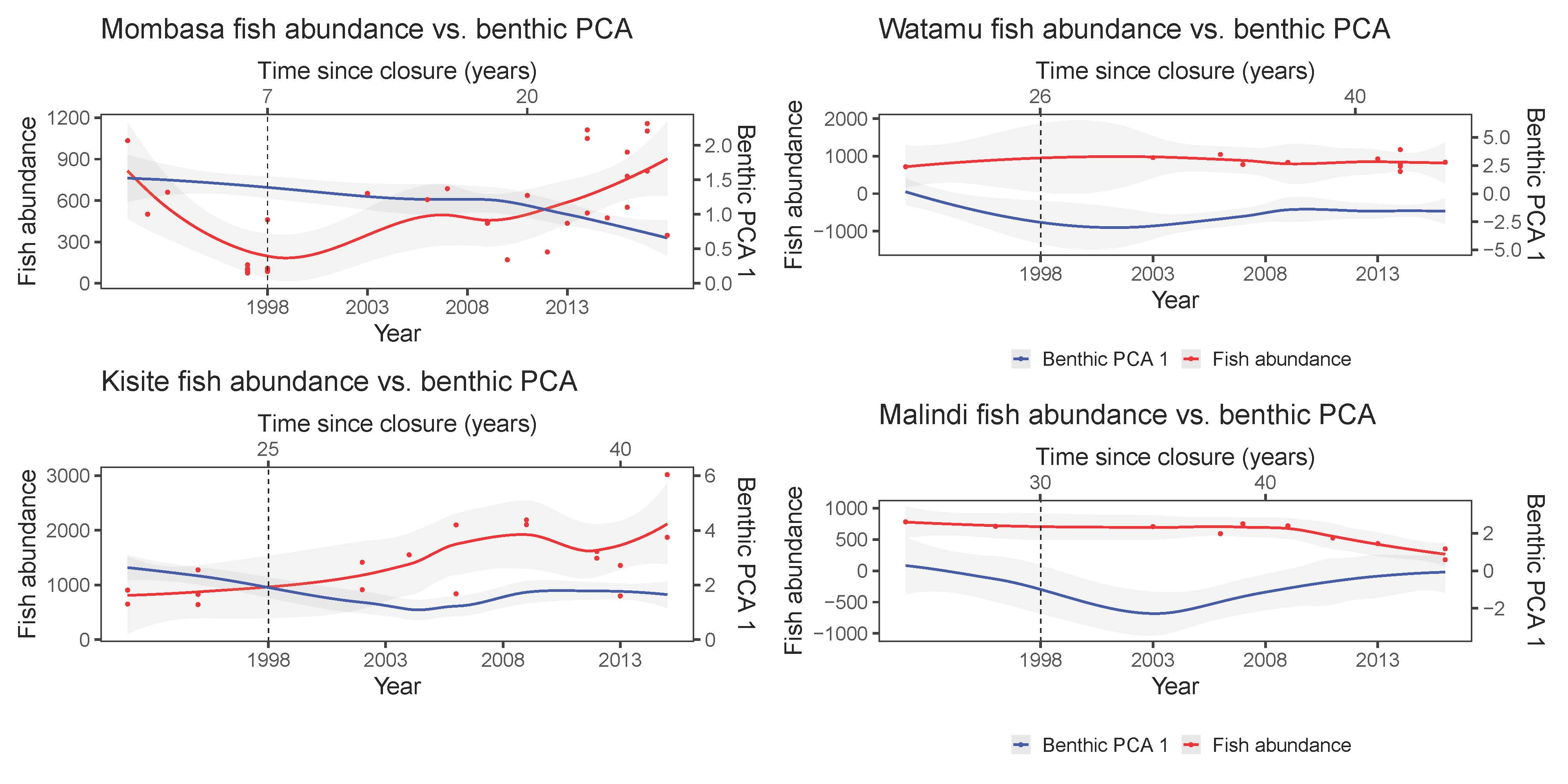


***Supplementary Figure S1.*** *Smoothed conditional means of benthic PCA (blue) and fish abundance (red) for each marine park. Dotted vertical line indicates 1998 bleaching event.*


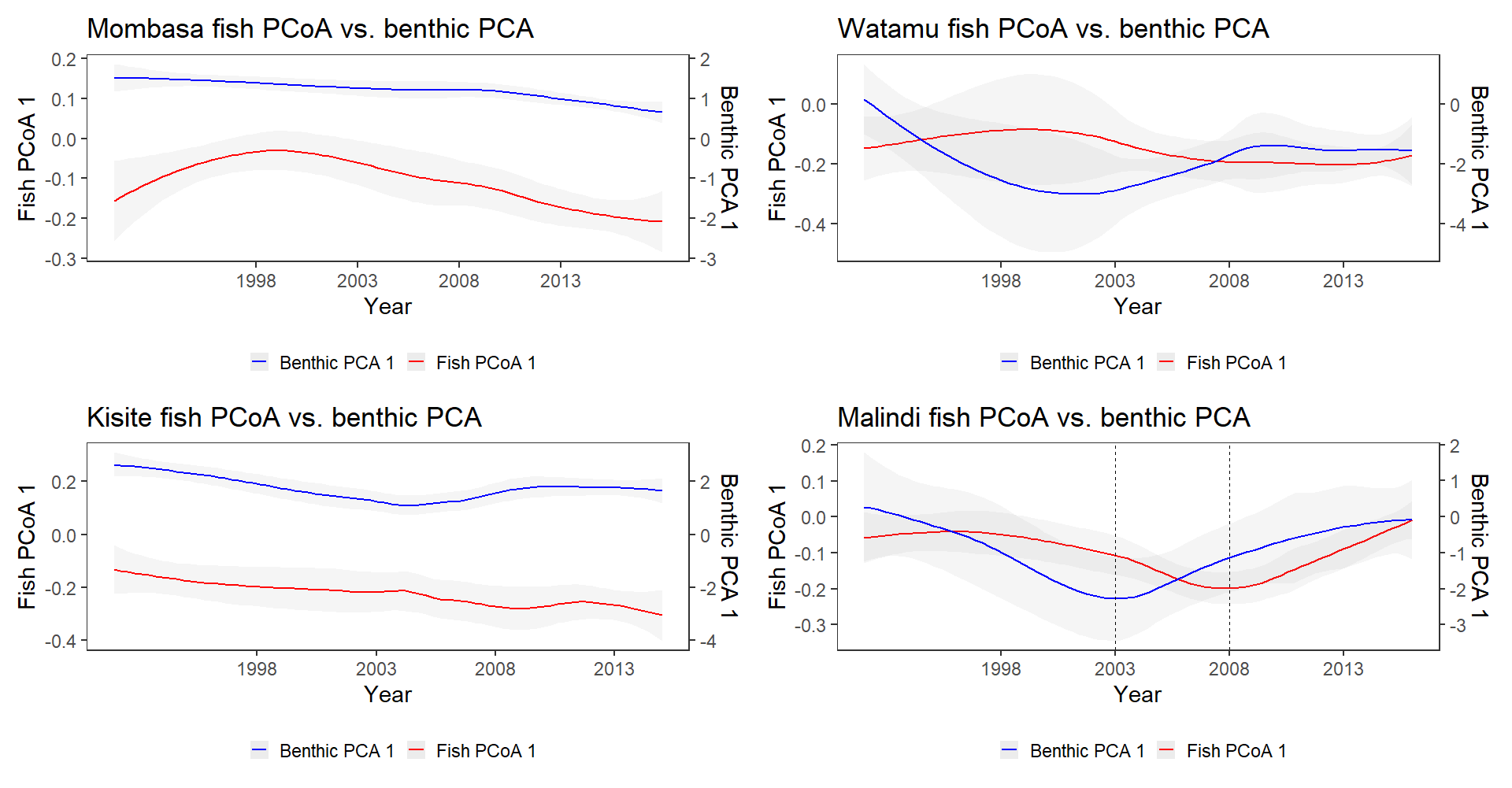


***Supplementary Figure S2.*** *Smoothed conditional means of benthic PCA (blue) and fish PCoA (red) for each marine park. Justification for removal of Malindi from the time-series. Figure shows Malindi fish PCoA follows the same pattern as benthic PCA and lags behind benthic PCA by 5 years.*


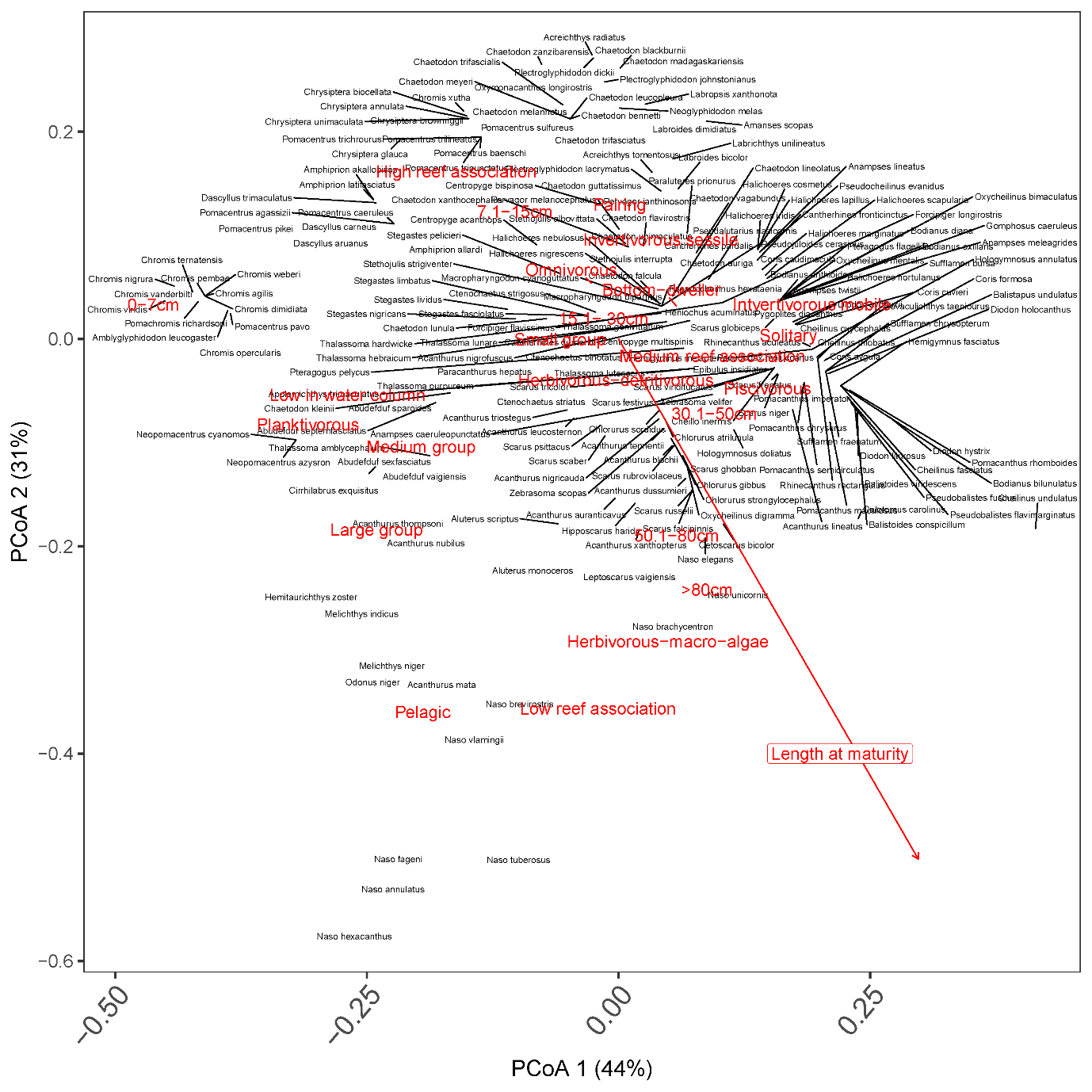


***Supplementary Figure S3.*** *PCoA of fish traits with species included in the ordination plot.*


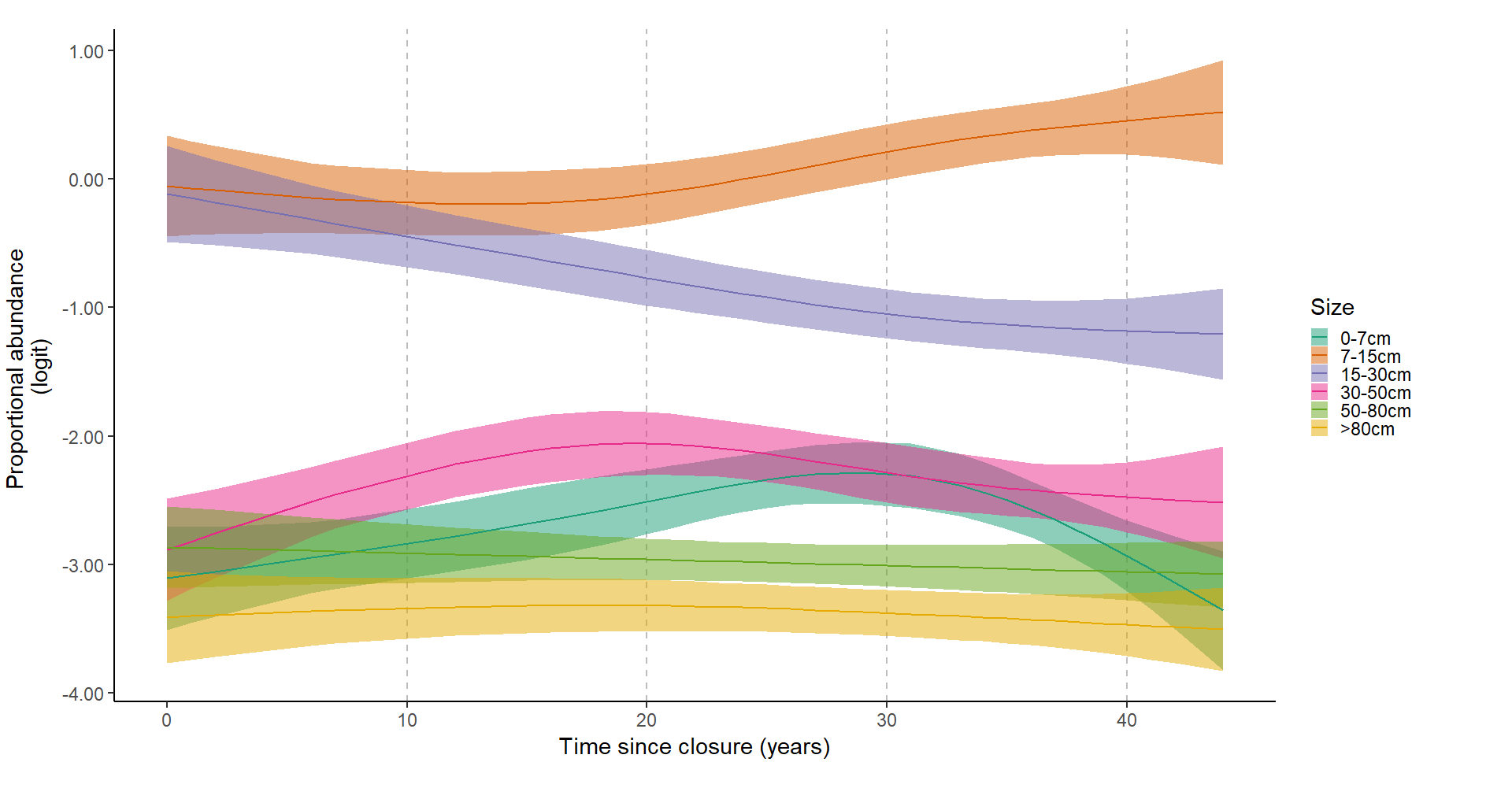


***Supplementary Figure S4.*** *Size class trait models over time since closure – sensitivity analysis removing Chromis dimidiata.*


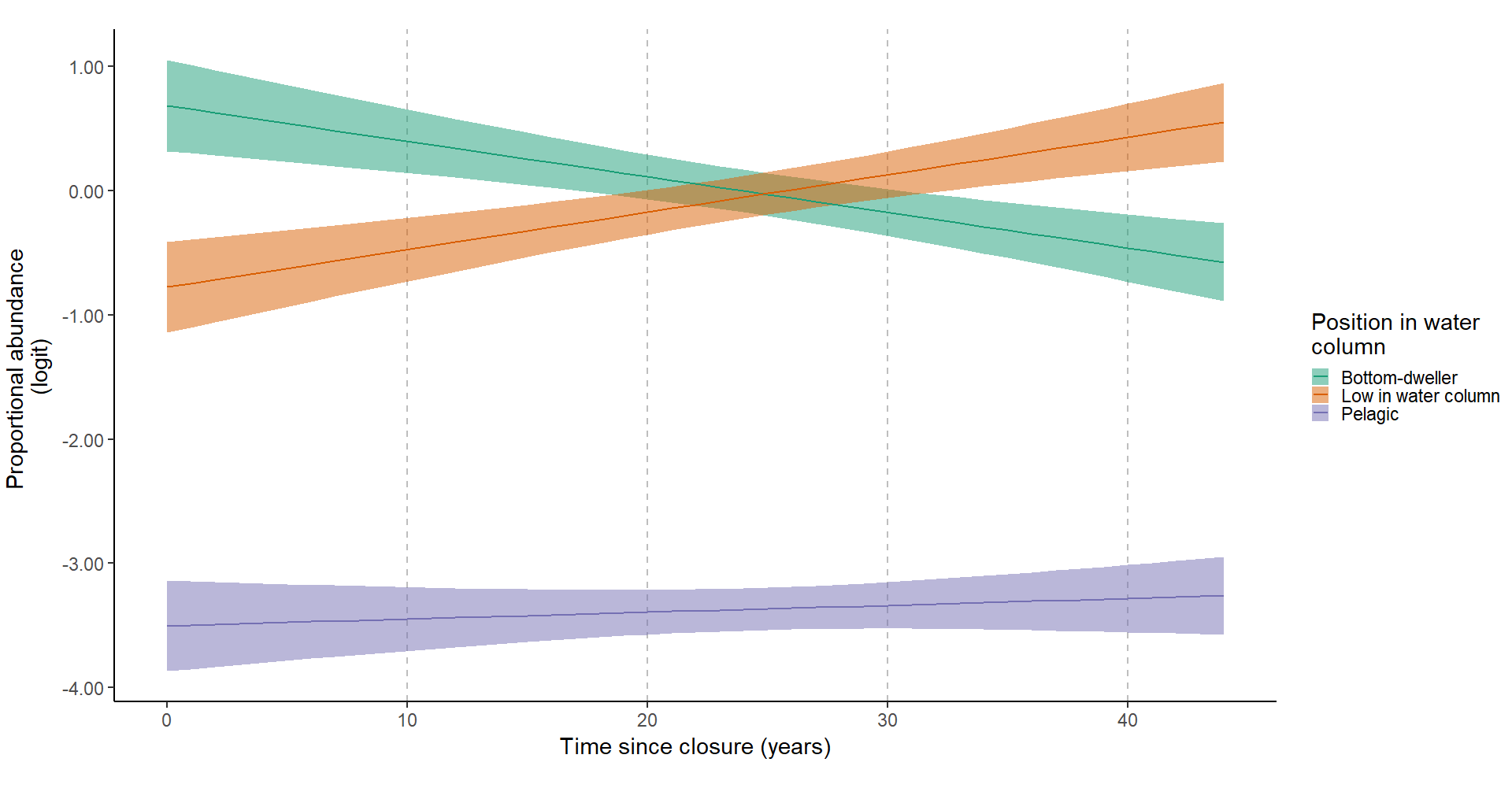


***Supplementary Figure S5.*** *Position in the water column trait models over time since closure. In this model we include points that are removed from the main manuscript figure 4.e as outliers. The points that are removed are from Mombasa Marine Park after 6-7 years of closure, where the relative abundance of species low in the water column is recorded as 100% or close to 100%. Because all of these points are in the same site/year, we removed them from the main manuscript to ensure that the overall trends did not reflect sampling biases in those specific surveys.*


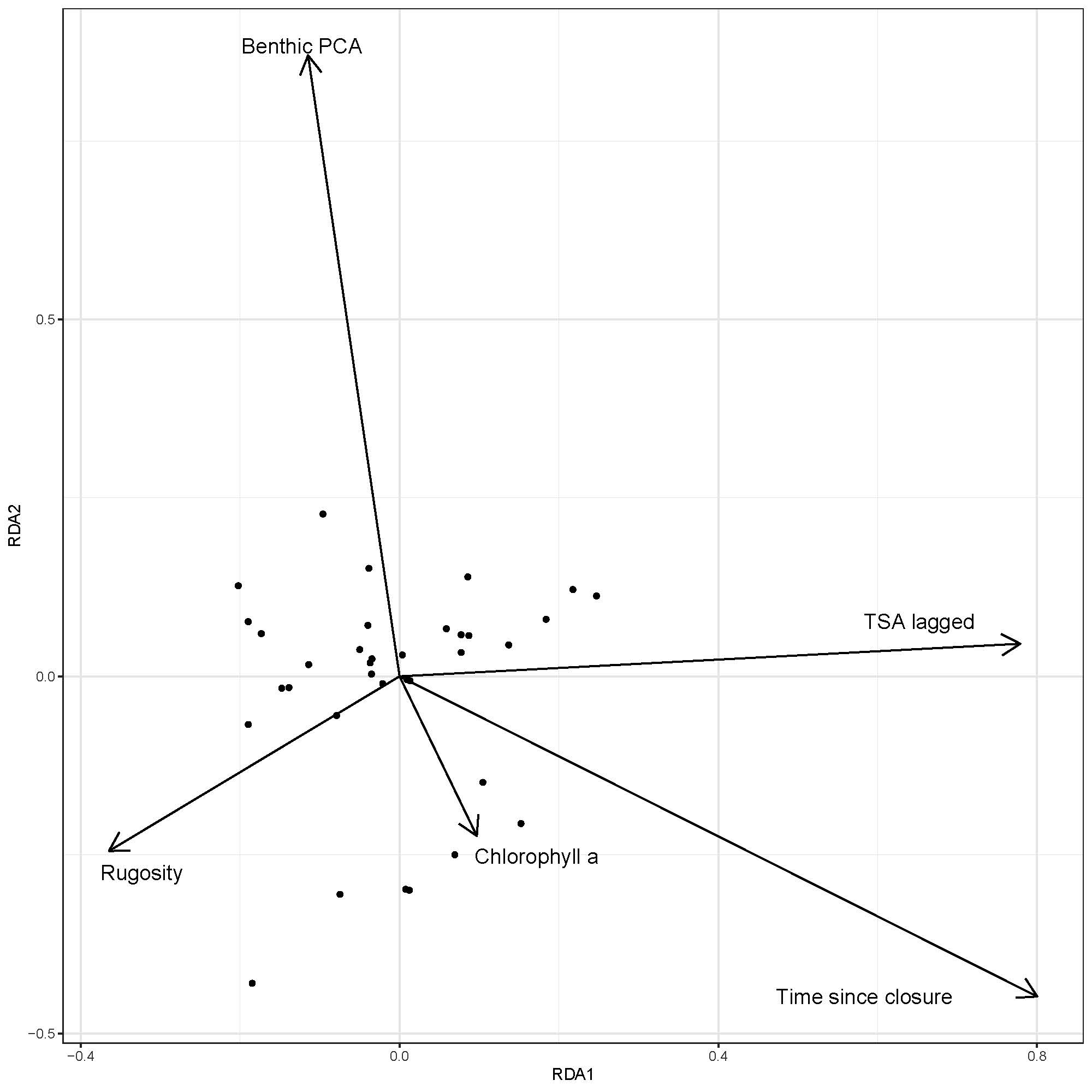


***Supplementary Figure S6.***  *Partial redundancy analysis (RDA) of environmental and management drivers (explanatory covariates) influencing fish community functional space****.***


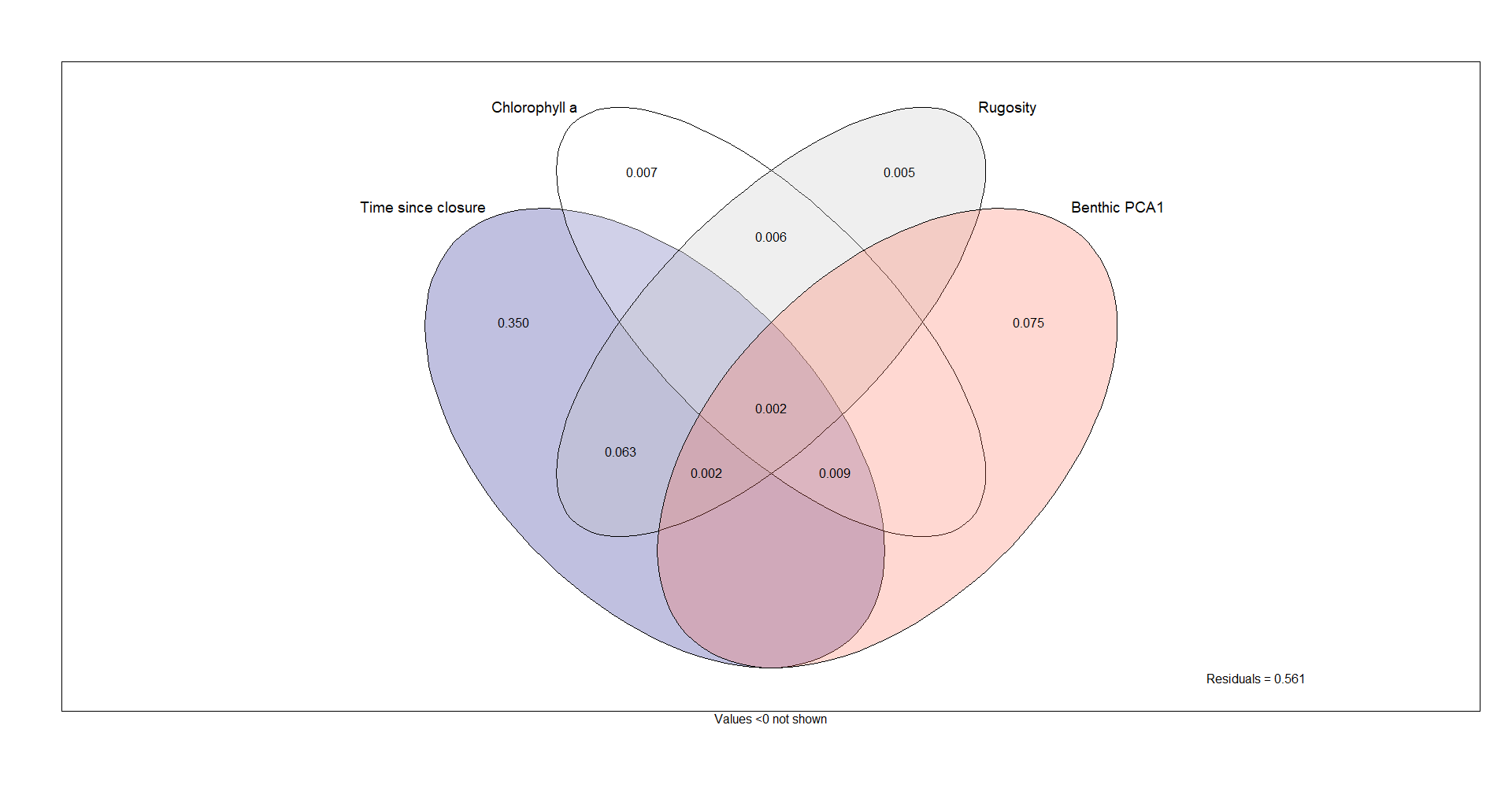


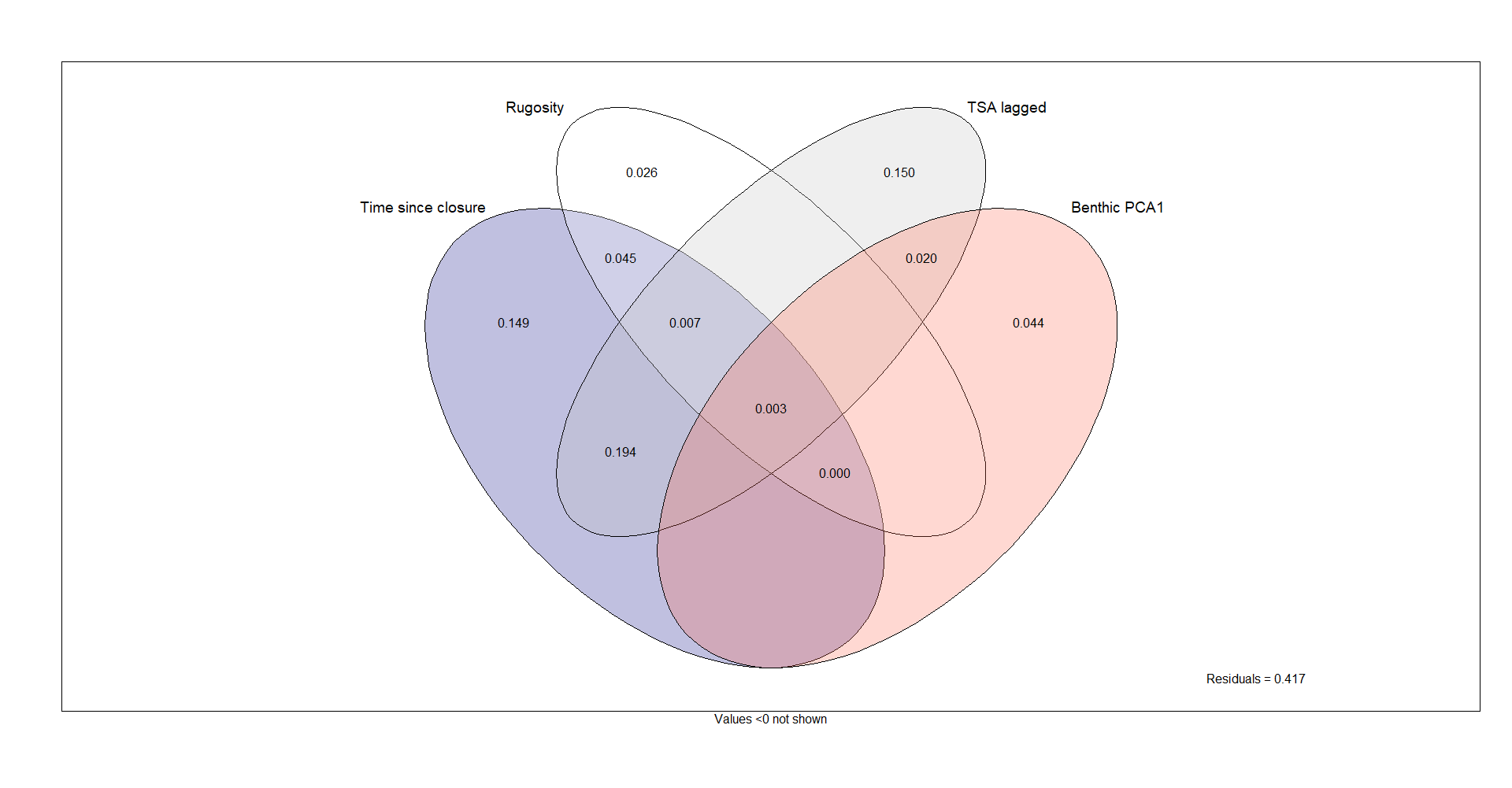


***Supplementary Figure S7.*** *Variance partitioning of environmental and management drivers (explanatory covariates) used in functional space models. Two plots are presented as only four covariates can be included in variance partitioning visualisations at a given time, and five covariates were used in the models.*


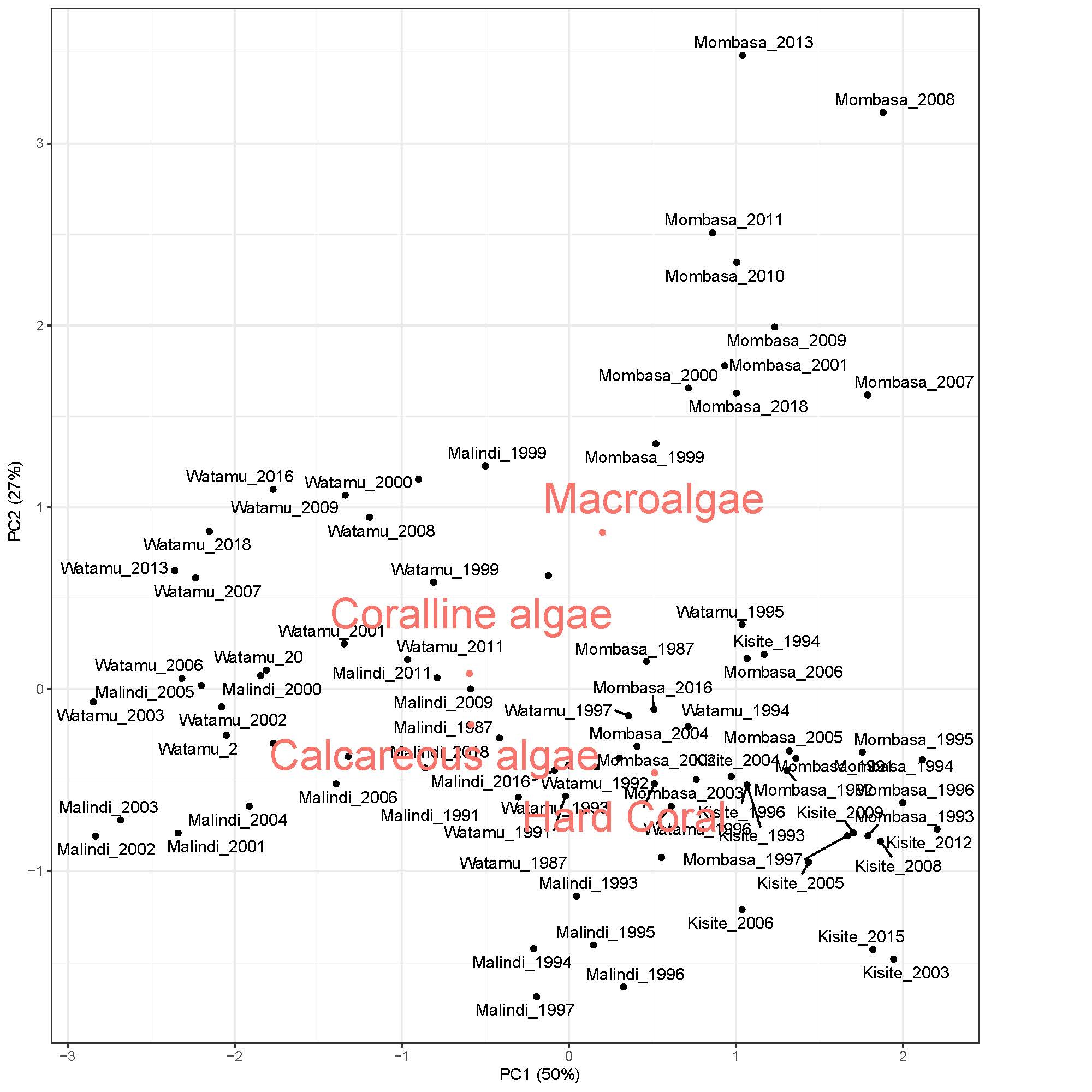


***Supplementary Figure S8.*** *Benthic PCA for all marine reserves.*

*
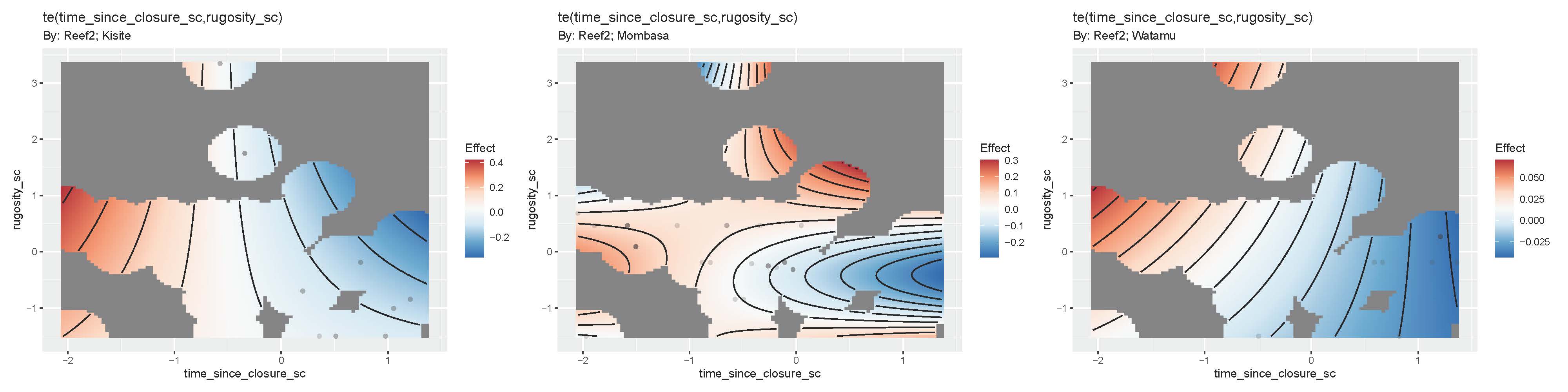

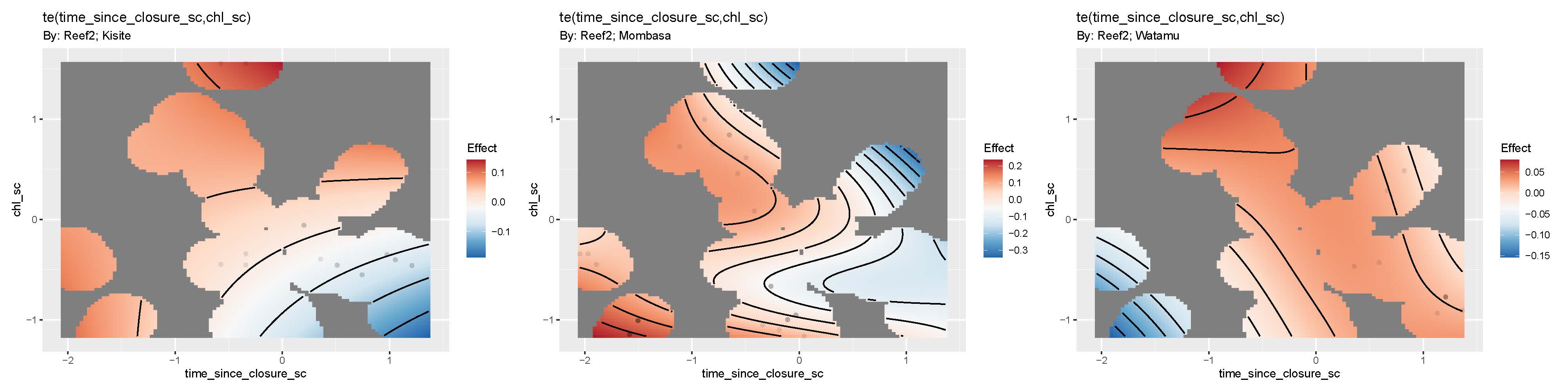

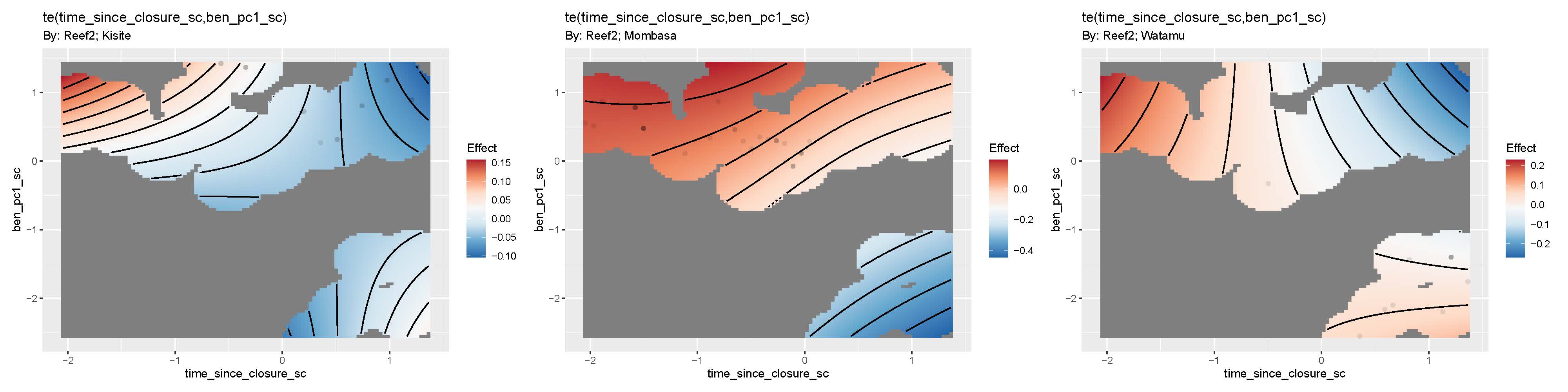

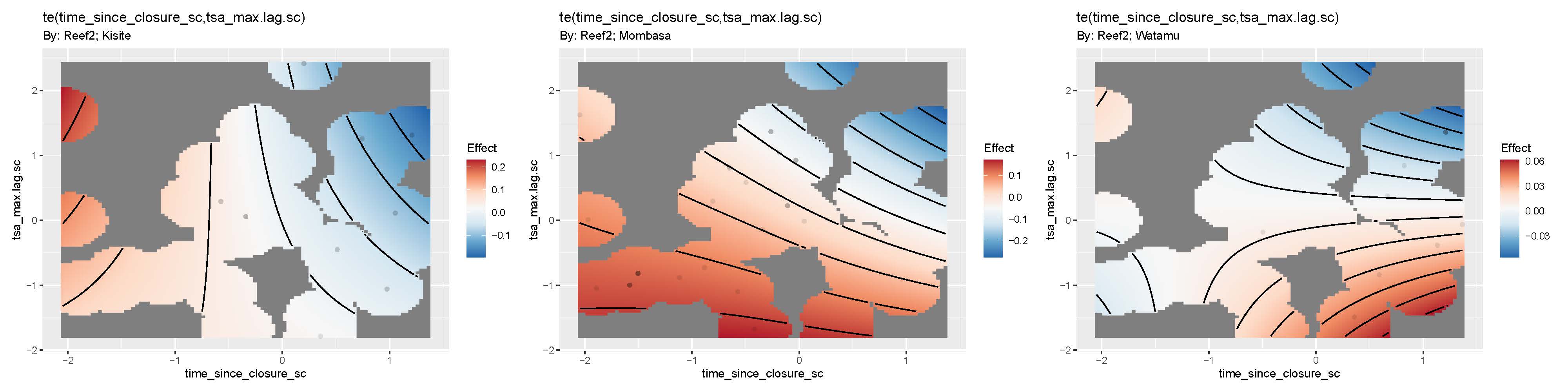
*

***Supplementary Figure S9.*** *Visualisation of tensor product splines of all covariates interacting with time since closure with PCoA axis 1 as the response variable, separated by marine reserve. Values outside of model prediction are in grey. Colour scale (from red to blue) corresponds to the effect of the variables on the PCoA axis.*

*
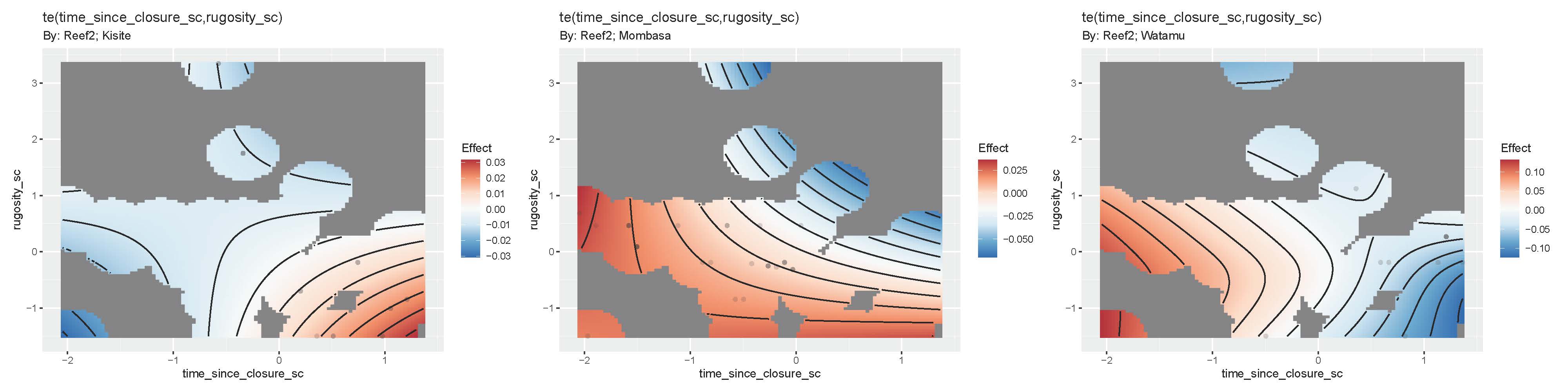

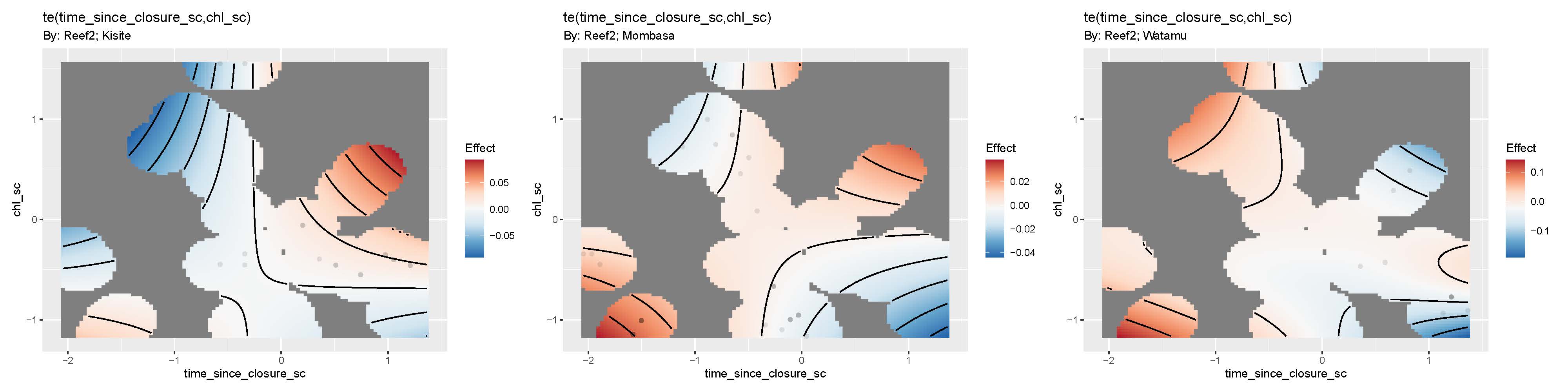

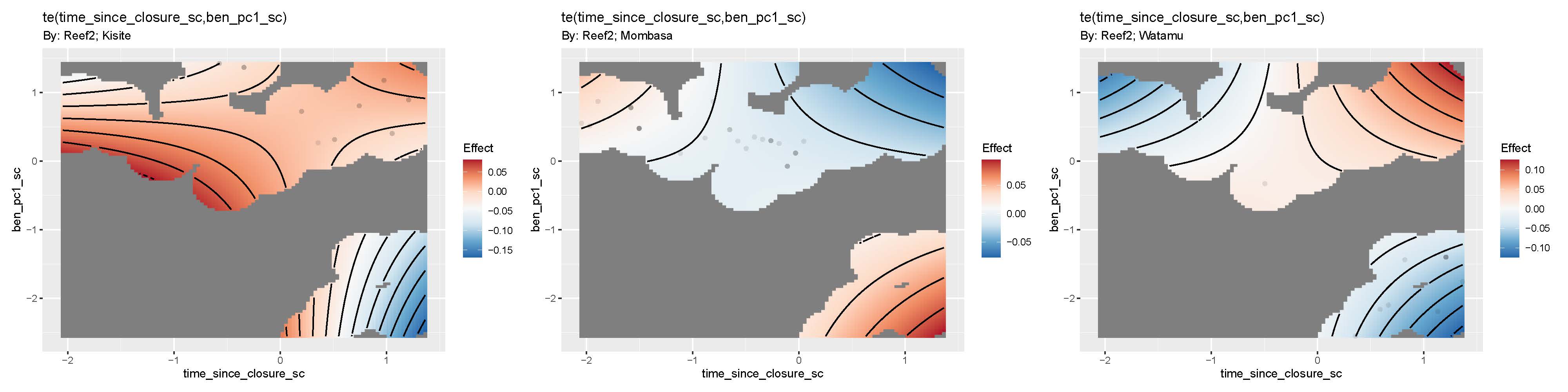

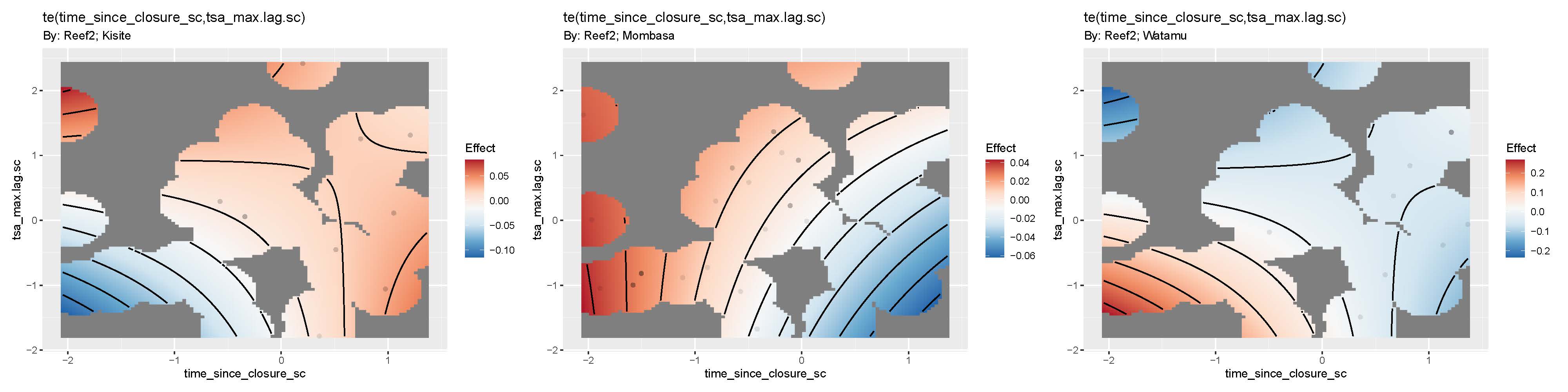
*

***Supplementary Figure S10.*** *Visualisation of tensor product splines all covariates interacting with time since closure with PCoA axis 1 as the response variable, separated by marine reserve. Values outside of model prediction are in grey. Colour scale (from red to blue) corresponds to the effect of the variables on the PCoA axis.*

**References**

1. McClanahan, T. R. & Graham, N. A. J. Marine reserve recovery rates towards a baseline are slower for reef fish community life histories than biomass. *Proceedings. Biol. Sci.* **282**, 20151938 (2015).

2. Robinson, J. P. W. *et al.* Fishing degrades size structure of coral reef fish communities. *Glob. Chang. Biol.* **23**, 1009–1022 (2017).

3. Allgeier, J. E., Valdivia, A., Cox, C. & Layman, C. A. Fishing down nutrients on coral reefs. *Nat. Commun.* **7**, 12461 (2016).

4. Bonaldo, R. M. & Bellwood, D. R. Size-dependent variation in the functional role of the parrotfish Scarus rivulatus on the Great Barrier Reef, Australia. *Mar. Ecol. Prog. Ser.* **360**, 237–244 (2008).

5. Lokrantz, J., Nyström, M., Thyresson, M. & Johansson, C. The non-linear relationship between body size and function in parrotfishes. *Coral Reefs* **27**, 967–974 (2008).

6. Nash, K. L., Graham, N. A. J. & Bellwood, D. R. Fish foraging patterns, vulnerability to fishing, and implications for the management of ecosystem function across scales. *Ecol. Appl.* **23**, 1632–1644 (2013).

7. Scharf, F., Juanes, F. & Rountree, R. Predator size-prey size relationships of marine fish predators: interspecific variation and effects of ontogeny and body size on trophic-niche breadth. *Mar. Ecol. Prog. Ser.* **208**, 229–248 (2000).

8. Jacob, U. *et al.* The Role of Body Size in Complex Food Webs: A Cold Case. *Adv. Ecol. Res.* **45**, 181–223 (2011).

9. Coleman, M. A. *et al.* Functional traits reveal early responses in marine reserves following protection from fishing. *Divers. Distrib.* **21**, 876–887 (2015).

10. Boaden, A. E. & Kingsford, M. . . Predators drive community structure in coral reef fish assemblages. *Ecosphere* **6**, art46 (2015).

11. Suchley, A. & Alvarez-Filip, L. Herbivory facilitates growth of a key reef-building Caribbean coral. *Ecol. Evol.* **7**, 11246–11256 (2017).

12. Bellwood, D. R., Hoey, A. S. & Hughes, T. P. Human activity selectively impacts the ecosystem roles of parrotfishes on coral reefs. *Proc. R. Soc. B Biol. Sci.* **279**, 1621–1629 (2012).

13. Ruttenberg, B. I., Adam, T. C., Duran, A. & Burkepile, D. E. Identity of coral reef herbivores drives variation in ecological processes over multiple spatial scales. *Ecol. Appl.* e01893 (2019). doi:10.1002/eap.1893

14. Michael, P. J., Hyndes, G. A., Vanderklift, M. A. & Vergés, A. Identity and behaviour of herbivorous fish influence large-scale spatial patterns of macroalgal herbivory in a coral reef. *Mar. Ecol. Prog. Ser.* **482**, 227–240 (2013).

15. Spitz, J., Ridoux, V. & Brind’Amour, A. Let’s go beyond taxonomy in diet description: Testing a trait-based approach to prey-predator relationships. *J. Anim. Ecol.* **83**, 1137–1148 (2014).

16. Fréon, P. & Misund, O. A. *Dynamics of pelagic fish distribution and behaviour : effects on fisheries and stock assessment*. (Fishing News Books, 1999).

17. Paramo, J., Gerlotto, F. & Oyarzun, C. Three dimensional structure and morphology of pelagic fish schools. *J. Appl. Ichthyol.* **26**, 853–860 (2010).

18. Magoulick, D. D. Effects of Predation Risk on Habitat Selection by Water Column Fish, Benthic Fish and Crayfish in Stream Pools. *Hydrobiologia* **527**, 209–221 (2004).

19. Rocha, L. A., Rocha, C. R., Baldwin, C. C., Weigt, L. A. & McField, M. Invasive lionfish preying on critically endangered reef fish. *Coral Reefs* **34**, 803–806 (2015).

20. Mason, N. W. H., Lanoiselée, C., Mouillot, D., Wilson, J. B. & Argillier, C. Does niche overlap control relative abundance in French lacustrine fish communities? A new method incorporating functional traits. *J. Anim. Ecol.* **77**, 661–669 (2008).

21. Floeter, S. R., Bender, M. G., Siqueira, A. C. & Cowman, P. F. Phylogenetic perspectives on reef fish functional traits. *Biol. Rev.* **93**, 131–151 (2018).

22. McClanahan, T. R. Response of the coral reef benthos and herbivory to fishery closure management and the 1998 ENSO disturbance. *Oecologia* **155**, 169–177 (2008).

23. Mellin, C., Aaron Macneil, M., Cheal, A. J., Emslie, M. J. & Julian Caley, M. Marine protected areas increase resilience among coral reef communities. *Ecol. Lett.* **19**, 629–637 (2016).

24. Cinner, J. E. *et al.* Gear-based fisheries management as a potential adaptive response to climate change and coral mortality. *J. Appl. Ecol.* **46**, 724–732 (2009).

25. Hixon, M. A. & Beets, J. P. Predation, Prey Refuges, and the Structure of Coral-Reef Fish Assemblages. *Ecol. Monogr.* **63**, 77–101 (1993).

26. Kaunda-Arara, B., Rose, G. A., Muchiri, M. S. & Kaka, R. Long-term Trends in Coral Reef Fish Yields and Exploitation Rates of Commercial Species from Coastal Kenya. *West. Indian Ocean J. Mar. Sci.* **2**, 105–116 (2004).

27. Wilson, S. K. *et al.* Exploitation and habitat degradation as agents of change within coral reef fish communities. *Glob. Chang. Biol.* **14**, 2796–2809 (2008).

28. Thorson, J. T., Munch, S. B., Cope, J. M. & Gao, J. Predicting life history parameters for all fishes worldwide. *Ecol. Appl.* **27**, 2262–2276 (2017).

29. King, J. R. & McFarlane, G. A. Marine fish life history strategies: applications to fishery management. *Fish. Manag. Ecol.* **10**, 249–264 (2003).

30. Taylor, B. M., Houk, P., Russ, G. R. & Choat, J. H. Life histories predict vulnerability to overexploitation in parrotfishes. *Coral Reefs* **33**, 869–878 (2014).

31. McClanahan, T. R. Multicriteria estimate of coral reef fishery sustainability. *Fish Fish.* **19**, 807–820 (2018).

32. Jennings, S., Greenstreet, S. P. R. & Reynolds, J. D. Structural change in an exploited fish community: a consequence of differential fishing effects on species with contrasting life histories. *J. Anim. Ecol.* **68**, 617–627 (1999).

33. Rochet, M.-J., Cornillon, P.-A., Sabatier, R. & Pontier, D. Comparative analysis of phylogenetic and fishing effects in life history patterns of teleost fishes. *Oikos* **91**, 255–270 (2000).

34. Darling, E. S. *et al.* Relationships between structural complexity, coral traits, and reef fish assemblages. *Coral Reefs* **36**, 561–575 (2017).

35. McClanahan, T. Coral reef fish communities, diversity, and their fisheries and biodiversity status in East Africa. *Mar. Ecol. Prog. Ser.* **632**, 175–191 (2019).

36. McLean, M. *et al.* A Climate-Driven Functional Inversion of Connected Marine Ecosystems. *Curr. Biol.* **28**, 3654-3660.e3 (2018).

37. Heenan, A., Williams, G. J. & Williams, I. D. Natural variation in coral reef trophic structure across environmental gradients. *Front. Ecol. Environ.* **18**, 69–75 (2020).

38. Riegl, B. *et al.* Water column productivity and temperature predict coral reef regeneration across the Indo-Pacific. *Sci. Rep.* **5**, 1–7 (2015).
